# Interactions between Obesity Status and Dietary Intake of Monounsaturated and Polyunsaturated Oils on Human Gut Microbiome Profiles in the Canola Oil Multicenter Intervention Trial (COMIT)

**DOI:** 10.3389/fmicb.2016.01612

**Published:** 2016-10-10

**Authors:** Shuaihua Pu, Hamidreza Khazanehei, Peter J. Jones, Ehsan Khafipour

**Affiliations:** ^1^Department of Human Nutritional Sciences, University of Manitoba, WinnipegMB, Canada; ^2^Richardson Centre for Functional Foods and Nutraceuticals, University of Manitoba, WinnipegMB, Canada; ^3^Gut Microbiome Laboratory, Department of Animal Science, University of Manitoba, WinnipegMB, Canada; ^4^Department of Medical Microbiology and Infectious Diseases, University of Manitoba, WinnipegMB, Canada

**Keywords:** monounsaturated fatty acid (MUFA), polyunsaturated fatty acid (PUFA), blended oil, gut microbiota, BMI

## Abstract

Long-term dietary fatty acid intake is believed to induce changes in the human gut microbiome which might be associated with human health or obesity status; however, considerable debate remains regarding the most favorable ratios of fatty acids to optimize these processes. The objective of this sub-study of a double-blinded randomized crossover clinical study, the canola oil multi-center intervention trial, was to investigate effects of five different novel oil blends fed for 30 days each on the intestinal microbiota in 25 volunteers with risk of metabolic syndrome. The 60 g treatments included three MUFA-rich diets: (1) conventional canola oil (Canola); (2) DHA-enriched high oleic canola oil (CanolaDHA); (3) high oleic canola oil (CanolaOleic); and two PUFA-rich diets: (4) a blend of corn/saﬄower oil (25:75) (CornSaff); and (5) a blend of flax/saﬄower oil (60:40) (FlaxSaff). Stool samples were collected at the end of each period. DNA was extracted and amplified for 16S rRNA gene pyrosequencing. A total of 17 phyla and 187 genera were identified. While five novel oil treatments failed to alter bacterial phyla composition, obese participants resulted in a higher proportion of Firmicutes to Bacteroidetes than overweight or normal weight groups (*P* = 0.01). Similarly at the genus level, overall bacterial distribution was highly associated with subjects’ body mass index (BMI). Treatment effects were observed between MUFA- and PUFA-rich diets, with the three MUFA diets elevating *Parabacteroides, Prevotella, Turicibacter*, and Enterobacteriaceae’s populations, while the two PUFA-rich diets favored the higher abundance of *Isobaculum*. High MUFA content feedings also resulted in an increase of *Parabacteroides* and a decrease of *Isobaculum* in obese, but not overweight subjects. Data suggest that BMI is a predominant factor in characterization of human gut microbiota profile, and that MUFA-rich and PUFA-rich diets impact the composition of gut microbiota at lower taxonomical levels mainly in obese subjects.

## Introduction

The prevalence of metabolic syndrome (MetS) is dramatically increasing worldwide (Mente et al., 2009), with major attention directed on examining effects of dietary fat types, and particularly the optimal ratio of saturated fatty acid (SFA), monounsaturated fatty acid (MUFA), and polyunsaturated fatty acid (PUFA). High amounts of n-6 PUFA, mainly linoleic acid (LA) replacing the SFA contents found in present Western diets, have been shown to prevent cardiovascular diseases (Kim et al., 2013). Other types of PUFA including n-3 PUFA-rich diets also resulted in weight loss in humans (Madsen et al., 2005). The Mediterranean diet has long been associated with decreased prevalence of obesity and diabetes, and increased longevity (Bergouignan et al., 2009), partly because of the favorable effects of its MUFA content from olive oil, mainly oleic acid (OA). Therefore, although current dietary recommendations suggest reductions in intakes of SFA, they fall short of providing the optimal ratios of unsaturated fatty acids needed to prevent MetS development. Novel modified oils, designed as sources of blended MUFA and PUFA contents, are expected to significantly improve risk factors of MetS, including central obesity, body mass index (BMI), blood pressure, impaired glucose tolerance, lipid profile, age and lifestyle (Alberti et al., 2005). Recent evidence suggest that the composition of gut microbiota may play a role in metabolism and adiposity, implicated in MetS risk (Ley, 2010).

Each individual houses a specific and diverse composition of gut microbiome, which potentially impacts their health. Differences in the gut microbiota patterns have been observed between normal weight and obese animals (Ley et al., 2005; Turnbaugh et al., 2008; Myles et al., 2014). In humans, the gut microbiota has also been reported to impact the physiological state of obesity (Turnbaugh et al., 2009a). Although the links between the composition of gut microbiota and specific conditions associated with obesity are not clearly understood, an increased ratio of two dominant bacterial phyla, Firmicutes to Bacteroidetes appears to associate with obese-type humans and further correlate to obesity and MetS development (Ley et al., 2006). Therefore, manipulation of gut microbiome composition in obese subjects may have potential therapeutic implications for maintaining human health.

The composition of the gut microbiota can change dramatically in response to long-term dietary intake as nutrients obtained through foods are essential to these bacteria. A recent study comparing the gut microbiota of children from Burkina Faso and Italy revealed that the ratio of two dominant phyla, Bacteroidetes and Firmicutes, were entirely different between the two groups (De Filippo et al., 2010). Here, differences in dietary patterns presumably contributed to the shifts in microbiota, which in turn influenced overall host physiology and metabolism. Studies on traditional Western diets rich in SFA but low in fiber content demonstrated that the diets rich in SFA possessed stronger effects than unsaturated fatty acids in shifting gut microbiota profiles toward those in obese individuals (de Wit et al., 2012; Liu et al., 2012). On the other hand, very few studies to date have investigated the impacts of novel vegetable oils rich in MUFA or PUFA on the gut microbiota in a longer-term human clinical trial.

Therefore, the cause-and-effect relationships between any shift of the gut microbiota, the obese state itself, and the diet remain unknown (Conterno et al., 2011).

Here, we hypothesized that healthy dietary oil treatments with diverse fatty acid compositions, particularly those rich in MUFA or PUFA, may influence the bacterial composition in human gut. Objectives of present study were to investigate (1) whether significant differences in the gut microbiota composition (as represented by the feces Krause and Khafipour, 2011) could be produced in response to MUFA or PUFA oil blends; (2) how the obese status influences the gut bacterial communities during the interventions; and (3) whether the change in gut microbiota composition is correlated with the change in biomarkers of MetS.

## Materials and Methods

### Clinical Design

The Canola Oil Multi-center Intervention Trial (COMIT) was conducted at Richardson Centre for Functional Foods and Nutraceuticals (RCFFN) at University of Manitoba, the Institute of Nutraceuticals and Functional Foods (INAF) at Laval University, and the Department of Nutritional Sciences at Pennsylvania State University (PSU) between September 2010 and March 2012. The intervention studies were reviewed and approved by institutional ethics boards in respective participating universities. Written consents were obtained from all subjects as prescribed by Research Ethics Boards at all clinical centers. The protocol of the present sub-study of COMIT was approved by Biomedical Research Ethics Board (BREB) at University of Manitoba. The clinical trial was registered with clinicaltrials.gov (NCT01351012).

The COMIT study was designed as a random, controlled, double-blind, crossover clinical trial on volunteers at risk of MetS, as previously described (Senanayake et al., 2014). Adult men and women with at least one of the following cardiovascular risk factors were recruited for the study: waist circumference ≥94 cm for men and ≥80 cm for women, triglyceride (TG): ≥ 1.7 mmol/L, HDL cholesterol (HDL-C): <1 mmol/L (men) or <1.3 mmol/L (women), blood pressure: ≥130 mmHg (systolic) and/or ≥ 85 mmHg (diastolic) and glucose: ≥ 5.5 mmol/L. Participants with thyroid disease, diabetes mellitus, kidney disease, liver disease, current smokers, or people drinking more than two alcoholic beverages per week were excluded from the study. During the trial, volunteers who took any medication known to affect endothelial function during the trial were released from protocol, but those who were taking blood pressure medication with a constant dose were included. All participants provided written consent before clinical trial started. The endpoint data for serum lipid variables [Total Cholesterol (TC), HDL-C, LDL Cholesterol (LDL-C), TG] for the 25 subjects included in the microbiome study were extracted from previously reported COMIT data (Jones et al., 2014), reanalyzed (**Supplementary Figure S7**) and reinterpreted in the context of their correlations with microbial changes evaluated in this research.

The five-phase randomized full-feeding study design provided subjects with a consistent, individual weight-maintaining diet with a fixed 7-day rotation isocaloric menu of three meals and two snacks a day, including 50% carbohydrate, 15% protein and 35% fat of total energy of 3000 Kcal per day. A daily intake of 60 g dietary oils was equally distributed to two identical sizes of beverage shakes at breakfast and supper. Five oil treatments included: (1) canola oil [Canola; 63% MUFA, 20% LA, 10% α-linolenic acid (ALA)]; (2) DHA enriched canola-oil [CanolaDHA; 64% MUFA, 13% LA, 6% docosahexaenoic acid (DHA)]; (3) high OA canola oil (CanolaOleic; 72% MUFA, 15% LA, 2% ALA); (4) a blend of corn oil/saﬄower oil (CornSaff; 18% MUFA, 69% LA); and (5) a blend of flax oil/saﬄower oil (FlaxSaff; 18% MUFA, 38% LA, 32% ALA) (**Table 1**). All five diets were low in SFA, and SFA was replaced with different combinations of unsaturated fatty acids. Three canola-based diets were rich in MUFA while two saﬄower oil blends were high in n-3 PUFA or n-6 PUFA. To compare the impacts of MUFA-rich diets (Canola, CanolaDHA, and CanolaOleic) with those of PUFA-rich diets (CornSaff and FlaxSaff) on the gut microbiota, a contrast analysis was performed. Comparisons of subgroups between CanolaDHA and CanolaOleic, and CornSaff and FlaxSaff were also conducted. All the meals and shakes were prepared fresh in the metabolic kitchen. Participants were instructed to visit the research site and consume at least one meal containing one dose of treatment beverage (usually breakfast) under the supervision of the clinical staff in the cafeteria on weekdays, while the remaining meals and weekend proportions were packed for off-site consumption. No outside food was allowed to consume during the intervention period. Compliance was assessed by clinical staff by the rate of completion of meals provided under supervision as well as by the presence of food not consumed in their returned meal bags packed for off-site consumption. Both participants and study coordinators were blinded to the treatments. Each treatment phase lasted 30 days in duration and separated with 4 weeks washout periods.

**Table 1:** Fatty acid composition of five dietary oil treatments^1^ (g) consumed at 60 g/d (35% of energy intake based on 3000 kcal/d).

Fatty acids	Canola	CanolaDHA	CanolaOleic	CornSaff	FlaxSaff
	
			g (% of energy)		
SFA^2^					
C12:0	0.05	0.04	0.04	0.00	0.00
C14:0	0.04	0.47	0.04	0.00	0.00
C16:0	2.44	3.15	2.20	3.52	2.94
C17:0	0.04	0.10	0.05	0.01	0.00
C18:0	1.10	1.02	1.10	1.14	1.90
C20:0	0.39	0.34	0.39	0.06	0.00
C22:0	0.19	0.18	0.19	0.00	0.00
C24:0	0.11	0.09	0.11	0.00	0.00
**Total SFA**	4.36 (6.6)	5.39 (6.9)	4.12 (6.5)	4.73 (6.7)	4.84 (6.8)
MUFA^3^					
C16:1	0.15	0.12	0.13	0.02	0.00
C17:1	0.07	0.10	0.12	0.00	0.00
C18:1	35.17	37.95	42.88	10.56	10.72
C20:1	0.73	0.62	0.72	0.02	0.00
C22:1	0.03	0.04	0.04	0.00	0.00
**Total MUFA**	36.15 (17.6)	38.84 (17.8)	43.89 (19.3)	10.60 (9.5)	10.72 (9.6)
PUFA^4^					
C18:2	11.72	7.65	8.84	41.61	22.48
C18:3	5.86	1.18	1.38	0.17	19.19
C20:4	0.00	0.04	0.00	0.00	0.00
C20:5	0.00	0.09	0.00	0.00	0.00
C22:5	0.00	1.42	0.00	0.00	0.00
C22:6,	0.00	3.48	0.00	0.00	0.00
**Total PUFA**	17.58 (9.1)	13.86 (8.0)	10.22 (6.9)	41.78 (16.3)	41.67 (16.3)

*^1^Dietary oil treatments are Canola, conventional canola oil; CanolaDHA, high oleic canola oil with DHA (85:15); CanolaOleic, high oleic canola oil; CornSaff, corn oil and saﬄower oil blend (25:75); FlaxSaff, flax oil and saﬄower oil blend (60:40). ^2,3,4^SFA, saturated fatty acid; MUFA, monounsaturated fatty acid; PUFA, polyunsaturated fatty acid.*

### Stool Collection

Twenty-five participants from RCFFN (Winnipeg, MB, Canada) arm of the COMIT trial agreed to provide 5 g stool samples at the end of each intervention phase for the present study (**Supplementary Tables S1** and **S2**). Subjects were instructed to collect their own samples from only one bowel movement in the privacy of their home the day before they visited RCFFN on either day 29 or day 30 of their endpoint visits. Subjects were directed to store fecal specimens immediately after collection at -20°C freezer until collector tubes were handed over to clinical coordinators at RCFFN. Fecal specimens subsequently labeled and stored at -80°C until further analysis.

### DNA Extraction

The frozen fecal specimens in collector tubes were wrapped in individual zipper bags and placed in a cooler with ice packs at RCFFN. After transport to the Gut Microbiome and Large Animal Biosecurity Laboratories at Department of Animal Science, University of Manitoba, fecal samples were thoroughly thawed and homogenized at room temperature. Genomic DNA was then extracted from approximately 250 mg fecal mass using ZR Fecal DNA Kit (D6010, Zymo Research Corp., Orange, CA, USA), which included a bead-beating step to lyse microbial cells, according to the manufacturer’s protocol. To match the concentration requirement for pyrosequencing, DNA quantity was determined under a Beckman DU/800 spectrophotometer (Beckman Coulter, Inc., Fullerton, CA, USA). Genomic DNA was normalized to achieve a concentration of 20 ng/μL and quality checked by PCR amplification of the 16S ribosomal RNA (rRNA) gene, 27f (5′-GAAGAGTTTGATCATGGCTCAG-3′) and 342r (5′-CTGCTGCCTCCCGTAG-3′) (Sepehri et al., 2007; Khafipour et al., 2009). Amplicons were verified by agarose gel electrophoresis.

### Pyrosequencing

Approximately 200 ng aliquots of high quality, inhibitor-free genomic DNA were sent to the Research and Testing Laboratories (Lubbock, TX, USA) for bacterial rRNA gene tag-encoded GS FLX-Titanium amplicon pyrosequencing (Dowd et al., 2008). Briefly, a mixture of Hot Start, HotStar high-fidelity Taq polymerases, and Titanium reagents were used to perform a one-step PCR (35 cycles). Pyrosequencing was performed using a 454 GS FLX-Titanium Sequencing System (454 Life Sciences, Roche Company, Branford, CT, USA). The primers 28f (GAGTTTGATCMTGGCTCAG) and 519r (GTNTTACNGCGGCKGCTG) were utilized to target the variable regions V1–V3 of the bacterial 16S rRNA genes. The sequencing data are uploaded into the Sequence Read Archive of NCBI^1^ and are accessible through accession number SRR2959981.

### Sequence Classification and Diversity Analysis

Sequencing data, which are categorical data, were edited, transformed and classified as described previously (Li et al., 2012). In general, all failed sequence reads, low-quality sequence ends, tags, and non-bacterial ribosome sequences and chimeras were removed from the dataset. Using software mothur (version 1.30.2) (Schloss et al., 2009), the second round of sequence quality control and assignments of operational taxonomic unit (OTU) were performed. All sequences less than 200 bp or sequences having one or more ambiguous base or containing a homopolymer length more than 8 bp were excluded from the data set. High quality 16S rRNA gene sequences were identified and aligned using the comprehensive rRNA database Silva (Pruesse et al., 2007) to reduce the noise from pyrosequencing data. The remaining sequence position columns and sequences were used to build a distance matrix with a distance threshold of 0.15. Using the furthest neighbor algorithm with a cutoff of 95% similarity, these sequences were clustered to OTU.

Within community diversity (α-diversity) was conducted based on OTU counts to evaluate the biodiversity of the bacterial population in the fecal samples at the genus level. Richness indices, Chao1 and abundance based coverage estimation (ACE), Shannon index and Simpson diversity were calculated to estimate the number of OTU that were present in each individual sample. Appropriate statistical considerations were used to exclude samples with extremely high or low diversity as outliers. A rarefaction curves, which allows the calculation of sequence richness, were also performed for five treatment groups and BMI groups using mothur (Schloss et al., 2009) based on randomly re-sampling the pool of sequence numbers.

To evaluate the differences in community composition among different treatments and BMI populations, β-diversity was measured by calculating the Bray-Curtis distance (Clarke et al., 2006). Principal coordinate analysis (PCoA) was applied on resulting distance matrices to generate two-dimensional plots using PRIMER v6 software (Warwick and Clarke, 2006). Permutational multivariate analysis of variance (PERMANOVA) was performed to assess significant differences of β-diversity among the treatments and BMI groups on the basis of distance measures using permutation methods (Anderson, 2005).

### Statistical Analysis

Based on the outcomes from mothur, the phyla and genera in relatively low abundance (under 0.1% of community) were removed from further analysis. SAS version 9.2 (SAS Institute Inc., Cary, NC, USA) was used for data analysis. Normality of residuals for α-diversity indices was tested by Kolmogorov–Smirnov tests and histograms were used to test the Gaussian nature of the dataset. The significance of differences between treatments was analyzed using mixed model with treatment followed by Tukey adjustment.

Normal distribution was tested by Kolmogorov–Smirnov tests and histograms, and Box-Cox power transformation macro was used if necessary when analyzing statistical differences among treatments or contrasts at phylum and genus levels. Depending on the distribution of the residuals, MIXED or GLIMMIX models in SAS with mixed-effect analysis of variance with treatment as fixed effect, and subject as a random effect followed by Tukey adjustment, were conducted to estimate the significant differences. Differences between treatments were considered significant at *P* < 0.05 while trends were observed at *P* < 0.1.

The non-parametric Spearman correlation matrix was generated and Spearman’s ρ and *P*-values were calculated to investigate the correlations between fecal microbiome and serum lipid profiles using the PAST software (PAleontological Statistics; Hammer et al., 2001). The results were visualized over heatmap using core plot package of R (R Core Team, 2013). Taxa with relative abundance greater than 0.1% of community were included in the analysis. Strong and weak correlations were presented by dark and light colors, respectively. The Spearman’s Rho denotes whether the correlation between the taxa of interest and the selected parameter was positive (closer to 1, blue squares) or negative (closer to -1, red squares).

Partial least square discriminant analysis (PLS-DA) was performed on bacterial genera to identify the effects of dietary oil treatments on the bacterial community, according to our previously described analytical method with modifications (Li et al., 2012). For this analysis, data were scaled using Unit Variance in SIMCA-P+ 13.0 (Umetrics, Umea, Sweden). Cross-validation and permutation testing were conducted to determine significant PLS components in the optimal model. To avoid over parameterization of the model, a cut-off level was set for variable influence on projection (VIP) value in each model to improve *R*^2^ and *Q*^2^ with better prediction of the bacterial distribution. Significant differences across treatments or BMI groups were expressed in scatter and score plots, according to the PLS regression coefficients. Each individual genus 95% confidence interval determined the positive or negative correlations with dietary treatments or BMI groups.

## Results

### Sample Assessment by Pyrosequencing

After the 5-phase clinical trial, a total of 66 samples were collected from 25 subjects. Missing samples resulted from participants either forgetting to collect samples or failing to provide samples at expected sampling dates (**Supplementary Table S1**). In total, 209,490 sequences were first generated. The minimum, median and maximum lengths of sequences were 250, 393, and 698 bp, respectively. Screening, filtering, and pre-clustering processes resulted in 142,887 sequences.

### Alpha and β-Diversity Analyses

**FIGURE 1:**
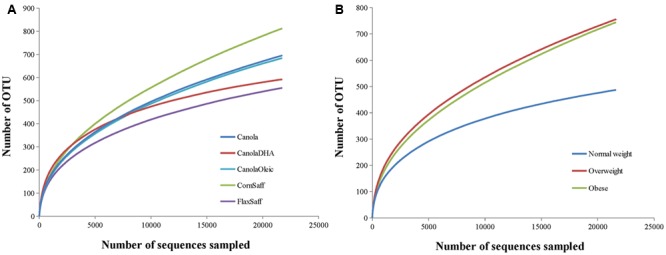
**(A,B)** Rarefaction analysis for the observed species. The rarefaction curve is generated using Chao1 richness estimator according to oil treatments **(A)** or subjects’ body mass index (BMI; **B**). Samples have been rarified at an even depth of 21,000 sequences per oil treatment or BMI group.

Bacterial richness and diversity in individual samples under different oil treatments and BMI were calculated (**Table 2**). Oil treatments and BMI had no significant impact on Chao1, ACE, Shannon and Simpson indices of α-diversity (**Figure 1A** and **Table 2**). However, the rarefaction curves showed higher richness and diversity in overweight and obese subjects compared to the normal weight participants (**Figure 1B**). Similarity or differences in the gut microbiota among different oil treatments and BMI (β-diversity) were compared using PCoA and PERMANOVA analyses of Bray-Curtis distances (**Supplementary Figures S1**–**S6**). Comparisons included MUFA vs. PUFA (*P* = 0.84), normal weight vs. overweight (*P* = 0.11), normal weight vs. obese (*P* < 0.01), overweight vs. obese (*P* = 0.03), MUFA vs. PUFA within overweight group (*P* = 0.57), and MUFA vs. PUFA within obese group (*P* = 0.99). The β-diversity did not change among individual oil treatments.

**Table 2:** Summary statistics for sequences using 16S rRNA gene pyrosequencing.

Variables^2^	Mean results for indicated variables^1^					
	Sequence Number	OTU Number^3^	Coverage (%)	Richness^4^		Diversity^5^	
				Chao 1	ACE	Shannon	Simpson
Treatments							
Canola	2521	121	98.38	173.86	218.10	3.17	0.11
CanolaDHA	2497	112	98.98	126.19	129.81	3.47	0.07
CanolaOleic	2486	116	98.46	162.87	201.23	3.23	0.09
CornSaff	2174	139	97.63	232.30	296.89	3.43	0.08
FlaxSaff	2414	111	98.37	149.42	181.24	3.28	0.11
SEM		14.78	0.26	13.67	46.67	0.15	0.02
*P*-value		0.69	0.01	0.15	0.15	0.53	0.58
BMI groups							
Normal	2401	121	98.50	150.15	162.20	3.46	0.07
Overweight	2673	134	98.30	186.84	224.48	3.37	0.09
Obese	2277	111	98.39	164.07	206.67	3.24	0.09
SEM		12.41	0.25	26.72	41.11	0.13	0.02
*P*-value		0.27	0.88	0.65	0.66	0.49	0.71

*^1^Means are from statistical models based on 66 fecal samples across five treatments. ^2^Dietary oil treatments are Canola, conventional canola oil; CanolaDHA, high oleic canola oil with DHA (85:15); CanolaOleic, high oleic canola oil; CornSaff, corn oil and saﬄower oil blend (25:75); FlaxSaff, flax oil and saﬄower oil blend (60:40). BMI groups are normal, overweight, and obese subjects. ^3^OTU, operational taxonomic units. ^4^Based on Chao1 and abundance based coverage estimation (ACE) richness indices. ^5^Based on Shannon and Simpson diversity estimators.*

### Overall Gut Microbiota Composition

A total of 15 phyla were identified in all the samples from 99.8% of all sequences (**Supplementary Table S3**), of which four phyla were considered as abundant within the community (larger than 1%), including Firmicutes, Bacteroidetes, Actinobacteria, and Proteobacteria (**Table 3**). The abundance of Aquificae, Fusobacteria, and Verrucomicrobia were between 0.1 and 1% of community. The remaining eight phyla, which were in low abundance (under 0.1%) included Acidobacteria, Chrysiogenetes, Cyanobacteria, Deinococcus-Thermus, Nitrospira, Synergistetes, TM7, and Tenericutes. Overall, the phylum distribution did not fluctuate across the five oil treatments or among MUFA vs. PUFA groups. However, BMI status had an impact on the microbiota composition at the phylum level with obese group having greater proportion of Firmicutes (*P* = 0.02) compared to the combined group of normal and overweight subjects (data not shown). The average ratio of Bacteroidetes to Firmicutes was 0.15 across five diets with no difference among dietary interventions (data not shown).

**Table 3:** Relative abundances of bacterial phyla from 16S rRNA gene sequences.

Phyla	Percentages of sequences in treatments^1^					Mean^2^	SEM	*P*-value^3^	Contrasts (*P*-value)^4^		
	Canola	CanolaDHA	CanolaOleic	CornSaff	FlaxSaff				MUFA/ PUFA	CanolaDHA/ CanolaOleic	CornSaff/ FlaxSaff
	Above 1% of community									
Actinobacteria	2.85	3.23	2.75	2.92	1.97	2.80	0.16	0.28	0.32	0.88	0.38
Bacteroidetes	11.60	11.77	12.81	10.90	10.77	11.62	0.71	0.83	0.51	1.00	0.85
Firmicutes	82.37	82.23	80.60	83.08	85.18	82.51	0.81	0.75	0.21	1.00	0.98
Proteobacteria	1.96	1.42	2.24	1.65	1.22	1.74	0.23	0.68	0.17	0.83	0.99
	Between 0.1 and 1% of community									
Aquificae	0.58	0.63	0.56	0.82	0.26	0.59	0.12	0.79	1.00	0.99	0.81
Fusobacteria	0.26	0.25	0.38	^∗^	0.18	0.22	0.08	0.68	0.91	1.00	N/A
Verrucomicrobia	0.19	0.16	0.26	0.13	0.24	0.19	0.05	0.95	0.84	0.98	0.98

*^1^Dietary oil treatments are Canola, conventional canola oil; CanolaDHA, high oleic canola oil with DHA (85:15); CanolaOleic, high oleic canola oil; CornSaff, corn oil and saﬄower oil blend (25:75); FlaxSaff, flax oil and saﬄower oil blend (60:40). ^2^Means for each phylum under five treatments are individually reported. The Mean and SEM are also reported across five treatments. ^3^*P*-values adjusted by Tukey are significantly different at *P* < 0.05. ^4^The contrast of different treatment groups or comparison between selected treatment groups. MUFA/PUFA is the contrast of the combination of Canola, CanolaDHA, and CanolaOleic compared to the combination of FlaxSaff and CornSaff. CanolaDHA/CanolaOleic is the comparison between CanolaDHA and CanolaOleic. CornSaff/FlaxSaff is the comparison between CornSaff and FlaxSaff. *P-*values adjusted by Tukey are significantly different at *P* < 0.05. ^∗^Percentage of sequences below 0.1.*

At the genus level, 187 genera were determined using 16S rRNA gene pyrosequencing. Majority of sequences were identified at the genus (g.) level while some sequences could only be classified up to phylum (p.), class (c.), order (o.), or family (f.) levels. **Supplementary Tables S4** and **S5** present the distribution of taxa with a relative abundance >0.1% and <0.1% of community, respectively, among diets. The abundance of g. *Faecalibacterium* tended to differ across treatments (*P* = 0.06) where CanolaOleic feeding was associated with the highest level of *Faecalibacterium* and CanoalDHA feeding with the lowest level. Data also showed a tendency for higher proportion of g. *Parabacteroides* (*P* = 0.09) but lower proportion of g. *Isobaculum* (*P* = 0.08) when the three MUFA-rich diets compared to two PUFA-rich diets. Genus *Blautia* was observed to favor the CanolaOleic feeding compared to the CanolaDHA diet (*P* = 0.09). No differences were observed between n-6 PUFA-rich treatment CornSaff and n-3 PUFA-rich diet FlaxSaff.

**FIGURE 2:**
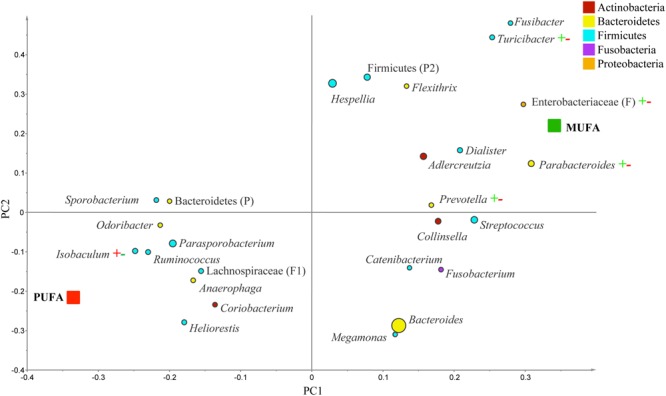
**Partial least square discriminant analysis (PLS-DA) loading plot based on the relative abundances of bacterial genera in the gut microbiota and their association with MUFA-rich or PUFA-rich diets.** The presenting genera are chosen at VIP cutoff of 1.0 and colored according to their corresponding phyla. The size of circles is indicative of taxa abundance. Taxa closer to MUFA or PUFA indicate positive association with either treatment group. The *R*^2^ (=0.43) and *Q*^2^ (=0.07) estimates were calculated based on three components. While majority of sequences were identified at the genus level (G), some could only be affiliated to phylum (P), class (C), order (O), or family (F) levels.

**FIGURE 3:**
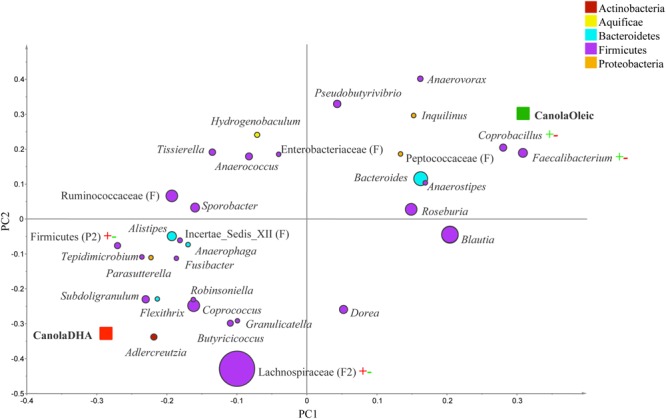
**Partial least square discriminant analysis loading plot based on the relative abundances of bacterial genera in the gut microbiota and their association with CanolaDHA or CanolaOleic diets.** The presenting genera are chosen at VIP cutoff of 1.0 and colored according to their corresponding phyla. The size of circles is indicative of taxa abundance. Taxa closer to CanolaDHA or CanolaOleic diets indicate positive association with either treatment group. The *R*^2^ (=0.78) and *Q*^2^ (=0.45) estimates were calculated based on three components. While majority of sequences were identified at the genus level (G), some could only be affiliated to phylum (P), class (C), order (O), or family (F) levels.

The PLS-DA analysis (given for the cutoff value of 1.0) showed further differences at the lower taxonomical levels. Genera *Parabacteroides, Prevotella, Turicibacter*, and f. Enterobacteriaceae were correlated to MUFA-rich diets, while g. *Isobaculum* was correlated to PUFA-rich diets (*R*^2^ = 0.43, *Q*^2^ = 0.07; **Figure 2**). Comparison between CanolaDHA and CanolaOleic indicated that CanolaDHA was correlated to f. Lachnospiraceae and p. Firmicutes, whereas CanolaOleic was associated with g. *Faecalibacterium* and *Coprobacillus* (*R*^2^= 0.78, *Q*^2^= 0.45; **Figure 3**). The comparison between two PUFA-rich diets showed CornSaff treatment had an impact on the populations of g. *Eggerthella, Slackia, Soehngenia, Anaerostipes, Robinsoniella, Phascolarctobacterium*, while FlaxSaff failed to have such significant impact (*R*^2^= 0.67, *Q*^2^= 0.22; data not shown).

### Gut Microbiota Profiles Within Individual Obese Status

**FIGURE 4:**
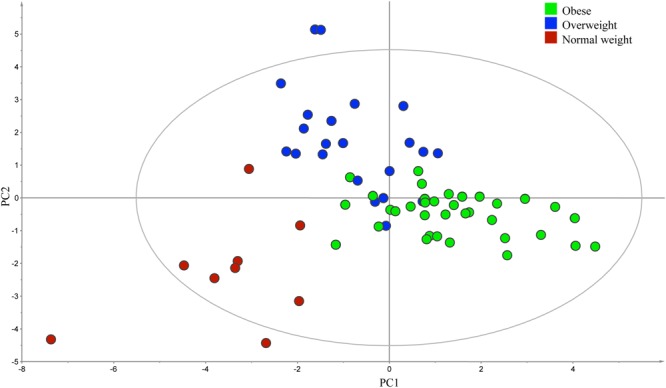
**Partial least square discriminant analysis score scatter plot of the gut microbiota comparing subjects with different body mass index (BMI).** The distribution of fecal samples (*n* = 66) indicated a significant difference in the composition of bacterial genera among obese (BMI > 30), overweight (BMI between 25 and 30), and normal weight (BMI < 25) groups. The *R*^2^ (=0.60) and *Q*^2^ (=0.32) estimates were calculated based on three components.

At the phylum level, a significantly higher proportion of Firmicutes was observed in obese group (BMI > 30) compared to the combined group of normal weight (BMI < 25) and overweight subjects (BMI between 25 and 30; *P* = 0.02; data not shown). At the genus level, PLS-DA analysis confirmed a significant difference in the composition of bacteria among three BMI phenotype groups (*R*^2^ = 0.60, *Q*^2^ = 0.32; **Figure 4**).

**FIGURE 5:**
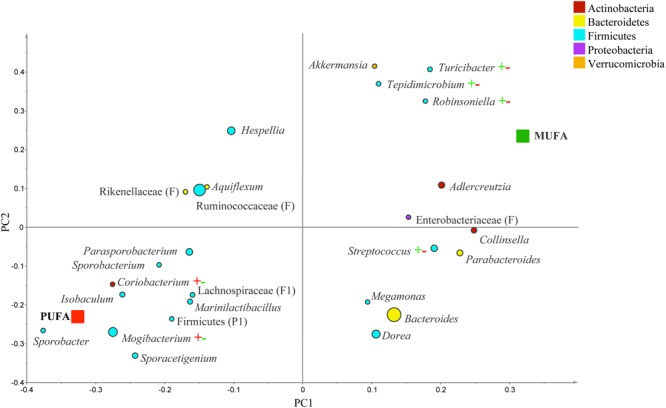
**Partial least square discriminant analysis loading plot based on the relative abundances of bacterial genera in the gut microbiota and their association with MUFA-rich or PUFA-rich diets in overweight population (BMI between 25 and 30).** The presenting genera are chosen at VIP cutoff of 1.0 and colored according to their corresponding phyla. The size of circles is indicative of taxa abundance. Taxa closer to MUFA or PUFA indicate positive association with either treatment group. The *R*^2^ (=0.69) and *Q*^2^ (=0.26) estimates were calculated based on three components. While majority of sequences were identified at the genus level (G), some could only be affiliated to phylum (P) class (C), order (O), or family (F) levels.

Since the number of subjects in the normal weight category was insufficient, the analysis on contrasts and subgroups only focused on two representative populations, overweight and obese groups. In overweight people, g. *Streptococcus, Tepidimicrobium, Robinsoniella*, and *Turicibacter* were correlated to MUFA-rich diets, while g. *Coriobacterium* and *Mogibacterium* were associated with PUFA-rich diets (*R*^2^ = 0.69, *Q*^2^= 0.26; **Figure 5**). In the comparison between CanolaDHA and CanolaOleic, g. *Adlercreutizia, Coriobacterium, Alistipes*, and *Robinsoniella* showed strong correlations with CanolaDHA while g. *Lactobacillus* was associated with CanolaOleic (*R*^2^ = 0.90, *Q*^2^= 0.60; data not shown). In the comparison between the two PUFA-rich diets, CornSaff was highly associated with g. *Adlercreutizia* while FlaxSaff was correlated with g. *Collinsella, Barnesiella, Streptococcus, Roseburia, Coprobacillus*, and family Peptostreptococcaceae (*R*^2^ = 0.98, *Q*^2^= 0.74; data not shown).

**FIGURE 6:**
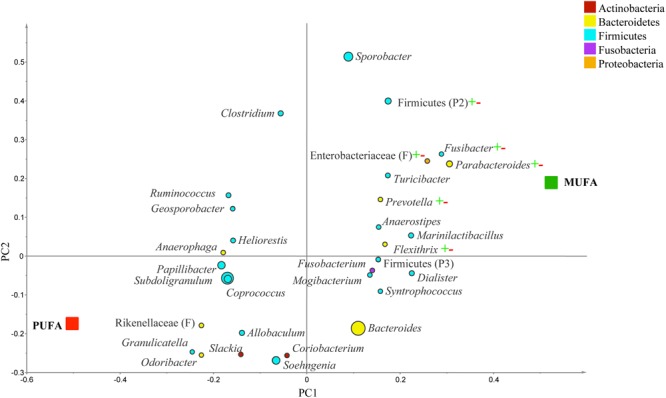
**Partial least square discriminant analysis (PLS-DA) loading plot based on the relative abundances of bacterial genera in the gut microbiota and their association with MUFA-rich or PUFA-rich diets in obese population (BMI > 30).** The presenting genera are chosen at VIP cutoff of 1.0 and colored according to their corresponding phyla. The size of circles is indicative of taxa abundance. Taxa closer to MUFA or PUFA indicate positive association with either treatment group. The *R*^2^ (=0.66) and *Q*^2^ (=-0.20) estimates were calculated based on three components. While majority of sequences were identified at the genus level (G), some could only be affiliated to phylum (P) class (C), order (O), or family (F) levels.

In obese subjects, g. *Parabacteroides, Prevotella, Flexithrix, Fusibacter*, f. Enterobacteriaceae, and p. Firmicutes were correlated to MUFA-rich diets, but no specific taxa was associated with PUFA-rich diets (*R*^2^ = 0.66, *Q*^2^= -0.20; **Figure 6**). In comparison of CanolaDHA and CanolaOleic, only g. *Parasutterlla* correlated with CanolaDHA (*R*^2^ = 0.91, *Q*^2^= 0.29; data not shown). Between the two PUFA-rich diets, g. *Collinsella, Hydrogenobaculum*, and *Parabacteroides* were impacted by the CornSaff, while g. *Clostridium* was correlated to the FlaxSaff diet (*R*^2^ = 0.98, *Q*^2^= 0.63; data not shown).

### Correlations between Serum Lipid Variables and Gut Microbiota Profiles

**FIGURE 7:**
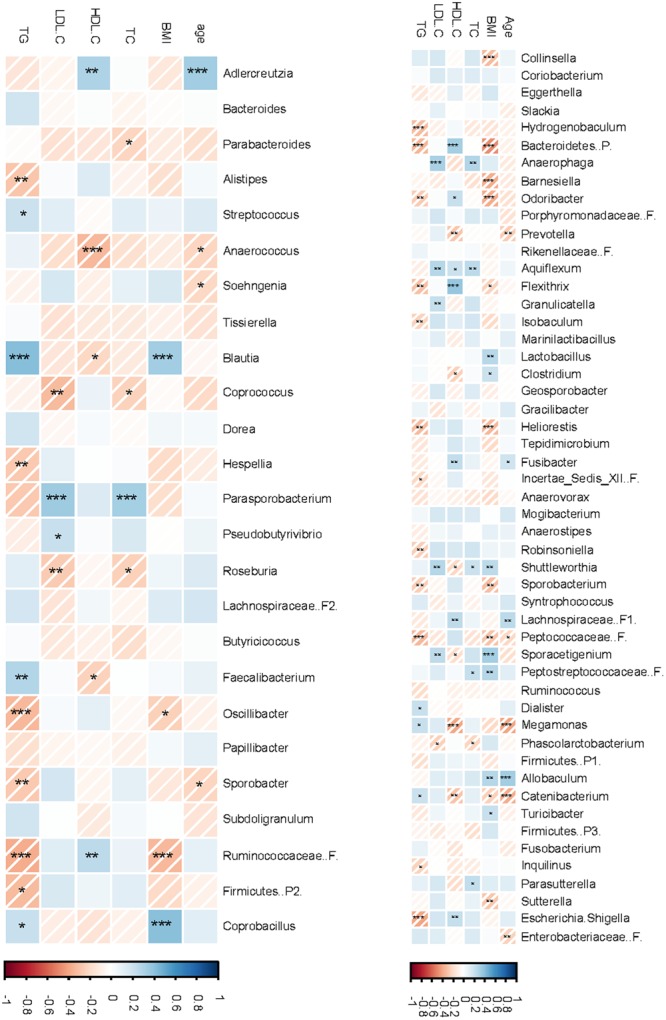
**Correlations between fecal microbiota compositions and serum lipid profiles.** The non-parameteric Spearman correlation coefficient and *P*-values were presented in a heatmap format. Strong and weak correlations are presented by dark and light colors, respectively. The intensity bar shows positive and negative correlation from blue to red, respectively. ^∗^*P* < 0.1; ^∗∗^*P* < 0.05; ^∗∗∗^*P* < 0.01. F.B, ratio of Firmicutes to Bacteroidetes.

The endpoint data for serum lipid variables of the 25 subjects included in this sub-study were extracted from previously reported COMIT data (Jones et al., 2014) and reanalyzed. All changes in lipid profiles followed the same trends of the entire study population (**Supplementary Figure S7**). The correlations between lipid profiles and bacterial phyla are presented in **Supplementary Table S6**. Across treatments, serum TG was negatively correlated with Aquificae (Spearman’s ρ = -0.27, *P* = 0.02) but positively correlated with Cyanobacteria (Spearman’s ρ = 0.24, *P* = 0.05). In contrast, LDL-C was positively correlated with p. Proteobacteria (Spearman’s ρ = 0.28, *P* = 0.01) and similar tendency was observed for TC (Spearman’s ρ = 0.21, *P* = 0.08). HDL-C, however, was positively correlated with Verrucomicrobia (Spearman’s ρ = 0.21, *P* = 0.08). In CanolaDHA treatment, TC levels trended to negatively correlate with Bacteroidetes (Spearman’s ρ = -0.51, *P* = 0.06) and positively correlated with Firmicutes (Spearman’s ρ = 0.55, *P* = 0.04), resulting in negative correlation trend with the ratio of Bacteroidetes to Firmicutes (Spearman’s Rho = -0.51, *P* = 0.06). In CornSaff treatment, TC levels were positively correlated with Bacteroidetes (Spearman’s ρ = 0.64, *P* = 0.02) and Bacteroidetes to Firmicutes ratio (Spearman’s ρ = -0.65, *P* = 0.02). The correlations between serum lipid variables and bacterial composition at the lower taxonomical levels are presented in **Figure 7**.

## Discussion

We investigated the impact of five different healthy unsaturated fatty acid oil treatments with a dietary fat content of less than 35% of energy intake on gut microbiota profiles of volunteers with risks of MetS. Our experimental diets included three MUFA-rich (Canola, CanolaDHA, and CanolaOleic) and two PUFA-rich (CornSaff and FlaxSaff) diets and were designed to maintain the body weight during the treatment periods. Major findings on post-treatment outcomes indicate that diet, metabolic profiles, obesity state, and individual responses are all contributing at different degrees to the shifts in the gut microbiota compositions. Overall, the impact of our healthy unsaturated fatty acid oil treatments on the microbiota composition was relatively minor at the phylum level and mainly associated with microbiota shifts at the lower taxonomical levels, whereas BMI contributed to a significant shift at the phylum level with greater Firmicutes and less Bacteroidetes in obese group compared to the combined group of normal and overweight subjects.

Several studies have investigated the effects of high fat diets mainly from SFA on the gut microbiota composition [8, 11, 14, 30, 31, 32]. Turnbaugh et al. (2008) showed that diet-induced obesity can quickly develop with altered microbiota profiles in mice fed prototypic Western diets with high SFA content. In a study comparing different types of high fat diets on the profile of gut bacteria in a mouse model, Liu et al. (2012) observed that consumption of a SFA-rich diet resulted in a significant decrease in the abundance of p. Bacteroidetes compared to either n-3 PUFA-rich or n-6 PUFA-rich diets. Similarly, a clinical study by Ley et al. (2006) described that the relative proportion of p. Bacteroidetes decreased in obese people compared with lean subjects, and this proportion also increased when the obese individual lost weight due to either carbohydrate-restricted or fat-restricted diets. Nielsen et al. (2007) reported that supplementation of fish oil for 1 month in human infants influenced the intestinal microbiota composition suggesting that dietary n-3 fatty acids may influence the bacterial adhesion to the intestinal mucosa, which in turn associates to the immune response and fat accumulation. Furthermore, Andersen et al. (2011) reported a significant increase in p. Bacteroidetes following the n-6 PUFA treatment. In the present study we did not have a control low fat or a control high fat treatment and the proportion of dietary fat content in all five treatments was less than 35% of total energy intake, which is the recommended level of fat intake in North America to maintain body weight (Grundy et al., 2002). As a result, our treatments were not considered high fat diet and we did not expect to see major shifts at the phylum level in the bacterial composition among n-3 PUFA (FlaxSaff), n-6 PUFA (CornSaff) and DHA-rich (CanolaDHA) treatments, or the combination of MUFA-rich vs. PUFA-rich diets. As expected the differences observed were at the lower taxonomical levels, for example MUFA-rich diets increased the populations of g. *Prevotella* and *Parabacteroides* within p. Bacteroidetes, and their impact was more pronounced in the obese people compared to normal and overweight subjects.

The individuals’ BMI has a major impact on gut microbiota. A study by Ley et al. (2005) used obese mice with ob/ob, lean ob/+, and wild-type siblings, and their ob/+ mothers to investigate the impact of obese genotypes and phenotypes on the gut microbiota. Results from a 16S rRNA gene sequence observation showed that ob/ob mice had a decreased abundance of Bacteroidetes and an increased proportion of Firmicutes. Authors also reported that the ratio of Bacteroidetes to Firmicutes dynamically reflects the overall weight condition in mice model. A follow-up study to extend these observations to humans showed that the relative proportion of Bacteroidetes is reduced in obese participants compared to the lean controls (Ley et al., 2006). However, it is very unlikely that the compositional shifts in Firmicutes to Bacteroidetes ratio can be solely responsible for development of obese phenotype. Other researchers, for example (Schwiertz et al., 2010), have failed to find similar trends when comparing the Firmicutes to Bacteroidetes ratio between the microbiota of obese and lean individuals, suggesting that the relationship between MetS, obesity, nutrition, and the microbiota is complex and multifactorial. Bacteroidetes genomes have a significantly higher level of functional diversity and, as demonstrated by the oft-cited study conducted by Turnbaugh et al. (2009a), the gut microbiome of human subjects from obese phenotype is functionally less diverse compared to the Bacteroidetes-rich gut microbiota of lean subjects. In the latter study, the majority of genes that were discriminating between the two phenotypes were annotated to carbohydrate, lipid and amino-acid metabolism pathways, with 75% of the obesity-enriched genes belonging to Actinobacteria and 42% of the lean-enriched genes to Bacteroidetes. These shifts in the functional properties of microbiota can change the energy harvesting capacities of the host via different mechanisms, for example, by altering the amount and profile of microbiota-derived short chain fatty acids (SCFAs), and/or, by modulating the expression of host genes that affect energy deposition in adipocytes (Bäckhed et al., 2007; Murphy et al., 2010). In the present study, subjects’ recruitment was based on the presence of at least one of the MetS risk factors including waist circumference, blood pressure, TG, HDL-C, and glucose levels. As a result, we didn’t have a balanced number of subjects at different BMI groups, and thus, we only observed a tendency of higher ratio of Bacteroidetes to Firmicutes in normal and overweight people compared to that of obese subjects. Nevertheless, when the obese states were classified as subgroups, the correlation coefficients were significantly improved indicating higher predictability of our models for detection of microbiota pattern in response to MUFA- or PUFA-rich diets.

Comparing the MUFA- and PUFA-rich diets, one interesting finding was the shift in g. *Parabacteroides* and *Isobaculum* populations suggesting that additional dietary OA can result in an increase in the population of *Parabacteroides* and a decrease in *Isobaculum*. When we chose the overweight and obese groups for subgroup analysis, contrasting MUFA vs. PUFA, a positive correlation between *Parabacteroides* and MUFA-rich diets was found to exist only in obese but not in overweight subjects. Further analysis comparing across CanolaOleic and CornSaff or FlaxSaff oil phases also showed consistent outcomes that this potential correlation with *Parabacteroides* was significant but was only observed in obese but not overweight volunteers. Thus, our study indicated that the MUFA-rich diets (high OA contents) can increase the population of *Parabacteroides* in human gut, and people with obese status may express a stronger response to such shift. Unfortunately, there is not much known about the function of *Parabacteroides* and *Isobaculum* and further investigation on the potential roles of these genera in development of obesity is needed.

Our results showed that g. *Faecalibacterium* population was higher in CanolaOleic treatment compared to CanolaDHA. Among different species of g. *Faecalibacterium, F. prausnitzii* is considered as an indicator of intestinal health due to its anti-inflammatory effects (Carlsson et al., 2013). Based on our findings, we can, therefore, hypothesize that additional intake of OA may increase the anti-inflammatory properties in human intestines by increasing the population of *F. prausnitzii* although further investigation is required.

The gut microbiome profile in humans is host-specific. Fecal communities in the human gut can change dramatically in response to new diets. Recent studies have shown that gut microbiome can rapidly and reproducibly respond to short-term dietary changes (David et al., 2014). These studies suggest that the human gut microbiome can rapidly switch between herbivorous and carnivorous functional profiles, which might be reflective of past selective pressures during human evolution (David et al., 2014). Turnbaugh et al. (2009b) using a humanized mice model showed that switching from a low-fat, plant polysaccharide-rich diet to a high-fat/high-sugar “Western” diet shifted the structure of the microbiota within a single day, changed the representation of metabolic pathways in the microbiome, and altered microbiome gene expression. The authors also indicated that the gut microbiota composition was stabilized 7 days following the dietary switch. As such, the 4-week dietary interventions used in this study should have been sufficient to stabilize diet-induced changes in the microbiota. It is clear now that each individual is relatively unique in terms of bacterial species of microbiota in their gut, no matter what obese state the individual belongs to, or what dietary oil treatments are provided. This may partly explain the variations in the microbiota profiles among the participants of our study and the variations in their responses to the dietary treatments.

Evidence showed dietary DHA oil intends to increase LDL-C levels in humans (Theobald et al., 2004; Jacobson et al., 2012), although the reason remains unclear. We observed similar elevated LDL-C levels after CanolaDHA treatment in the present study, which was significantly correlated with the increase in Firmicutes and the decrease in Bacteroidetes within this treatment. These observations are supported by a mouse study (Mujico et al., 2013), which reported that a diet supplemented with n-3 fatty acids (Eicosapentaenoic acid + DHA) significantly increased the abundance of Firmicutes and reduced the percentage of Bacteroidetes compared to a diet supplemented with OA. It can be speculated that the changes in metabolic parameters after DHA oil intake could be the result of interactions between gut microbiota and DHA metabolites potentially through the enterohepatic circulation of bile salts (Yokota et al., 2012; Sayin et al., 2013). However, our study was not designed to test this hypothesis and further investigation on the bile excretion analysis is required to proof or disproof this hypothesis.

All being said, our study also had several limitations. First, given an intervention trial period of more than 9 months of duration during which multiple samples were collected at different time points, some participants forgot to provide a fecal sample on the specific sampling day. Second, the small proportion of fecal samples might not be able to represent the total fecal materials. Even after the homogenization, the specimens could still produce large variations in the bacterial composition and, in addition, were limited to reflect the dynamic condition in the intestine. Third, the inclusion criteria of our study were based on the risks of MetS, but not the obese state. Given the lower numbers of participants with normal weight and overweight, the statistical analyses were not optimal to compare the direct variations among all participants.

In summary, the current study investigated the impact of five different healthy unsaturated fatty acid oil treatments on volunteers with risks of MetS. Results indicate that the human gut microbiota profiles strongly relate to the body obesity states, but also exhibit host-specificity. MUFA-rich and PUFA-rich diets failed to shift the composition of gut microbiota at the phylum level in a 30-day treatment period, but the populations of specific genera can slightly alter in response to the feedings due to unclear mechanisms. Further studies may clarify whether there is a cause-and-effect relationship between dietary fatty types perhaps through altered cholesterol and primary and secondary bile acid concentration, and shifts in human gut microbiota.

## Author Contributions

SP, PJ, and EK conceived and designed the experiment. SP and HK performed lab analyses. EK developed the bioinformatics and statistical models. SP and HK analyzed the data. SP, PJ, and EK wrote the manuscript.

## Conflict of Interest Statement

The authors declare that the research was conducted in the absence of any commercial or financial relationships that could be construed as a potential conflict of interest.

## Footnotes

1 http://www.ncbi.nlm.nih.gov/sra

## Supplementary Material

FIGURE S1 **Principal coordinates analysis (PCoA) of Bray-Curtis distance between the MUFA and PUFA oil treatments**.

FIGURE S2 **Principal coordinates analysis (PCoA) of the Bray-Curtis distance between the normal weight and overweight subjects**.

FIGURE S3 **Principal coordinates analysis (PCoA) of the Bray-Curtis distance between the normal weight and obese subjects**.

FIGURE S4 **Principal coordinates analysis (PCoA) of the Bray-Curtis distance between the overweight and obese subjects**.

FIGURE S5 **Principal coordinates analysis (PCoA) of the Bray-Curtis distance between the MUFA and PUFA oil treatments within overweight group**.

FIGURE S6 **Principal coordinates analysis (PCoA) of the Bray-Curtis distance between the MUFA and PUFA oil treatments within obese group**.

FIGURE S7 **Serum lipid profiles at the endpoint of five experimental diets.** Significant differences between treatments are highlighted within each parameter.

TABLE S1 **List of fecal samples collected from the COMIT study**.

TABLE S2 **Example of a 7-day rotation isocaloric menu during the intervention**.

TABLE S3 **Summary of sequenced OTUs at each taxonomic level**.

TABLE S4 **Phylogenetic composition of putative bacterial genera (above 0.1% of community) determined using 16S rRNA gene pyrosequencing**.

TABLE S5 **List of bacterial genera in low abundance (below 0.1% of community)**.

TABLE S6 **Correlation between bacterial phyla and metabolic parameters**.
