# Supplementary figures and images for: Interactions between Obesity Status and Dietary Intake of Monounsaturated and Polyunsaturated Oils on Human Gut Microbiome Profiles in the Canola Oil Multicenter Intervention Trial (COMIT)
Source: Front Microbiol. 2016 Oct 10;7:1612. doi: 10.3389/fmicb.2016.01612 (PMC5056191; doi:10.3389/fmicb.2016.01612)

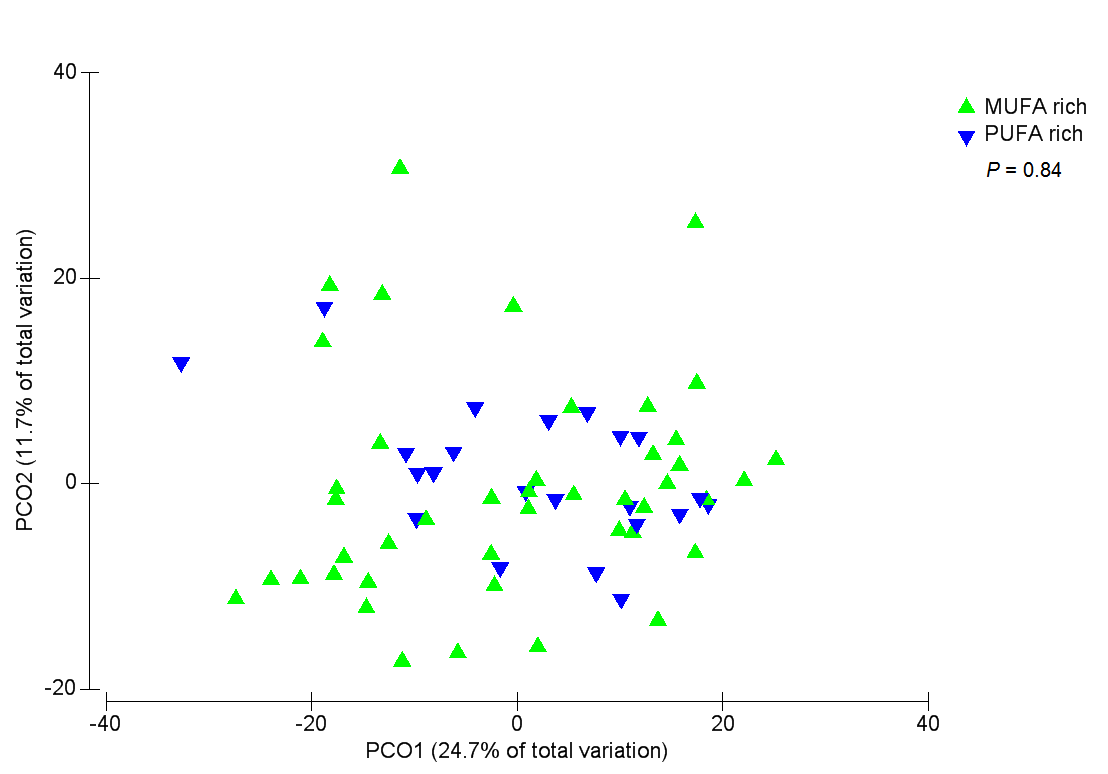

Supplement: FIGURE S1 — Principal coordinates analysis (PCoA) of Bray-Curtis distance between the MUFA and PUFA oil treatments. [file Image_1.TIF]

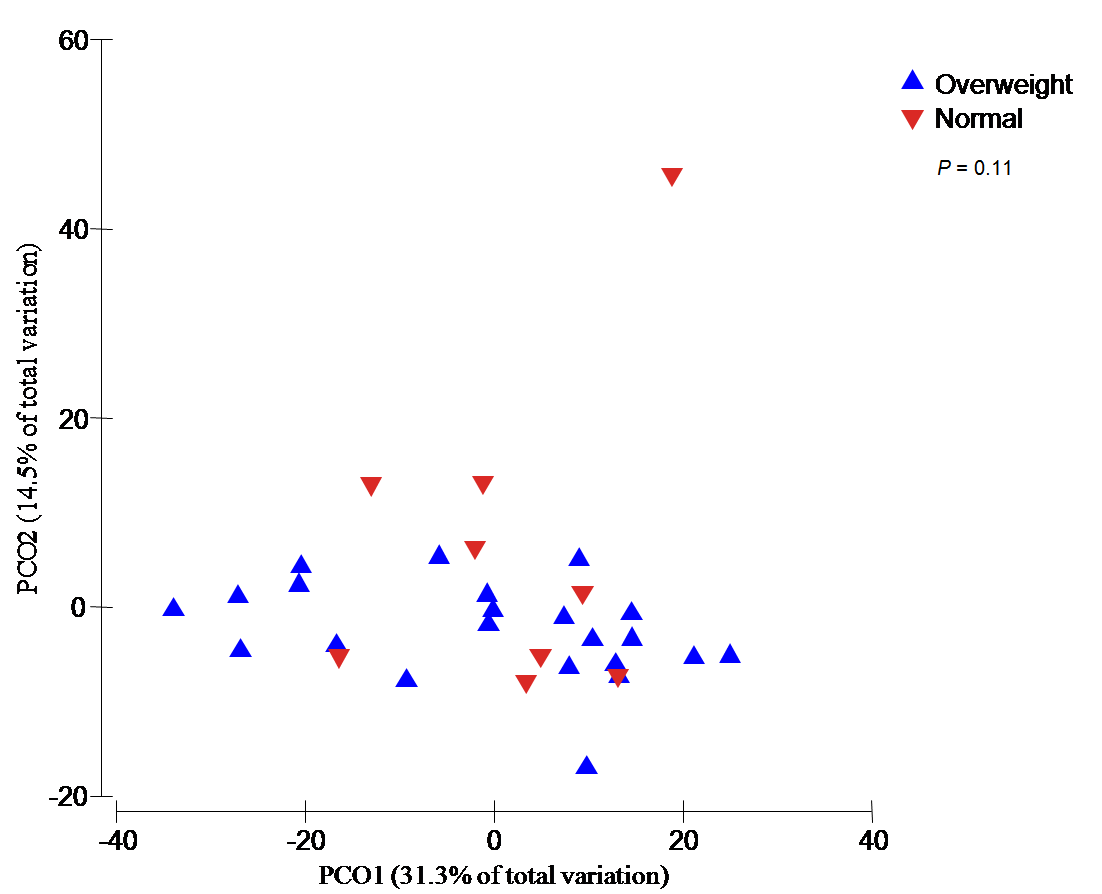

Supplement: FIGURE S2 — Principal coordinates analysis (PCoA) of the Bray-Curtis distance between the normal weight and overweight subjects. [file Image_2.TIF]

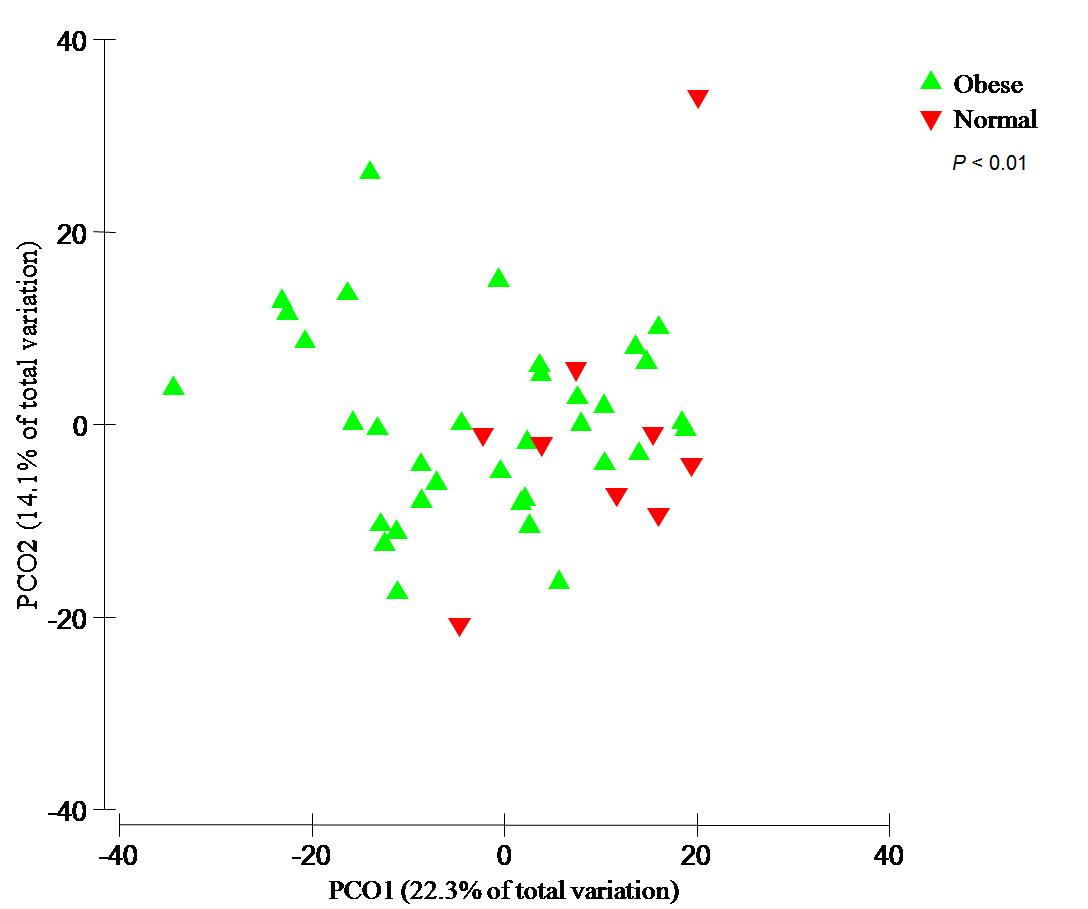

Supplement: FIGURE S3 — Principal coordinates analysis (PCoA) of the Bray-Curtis distance between the normal weight and obese subjects. [file Image_3.TIF]

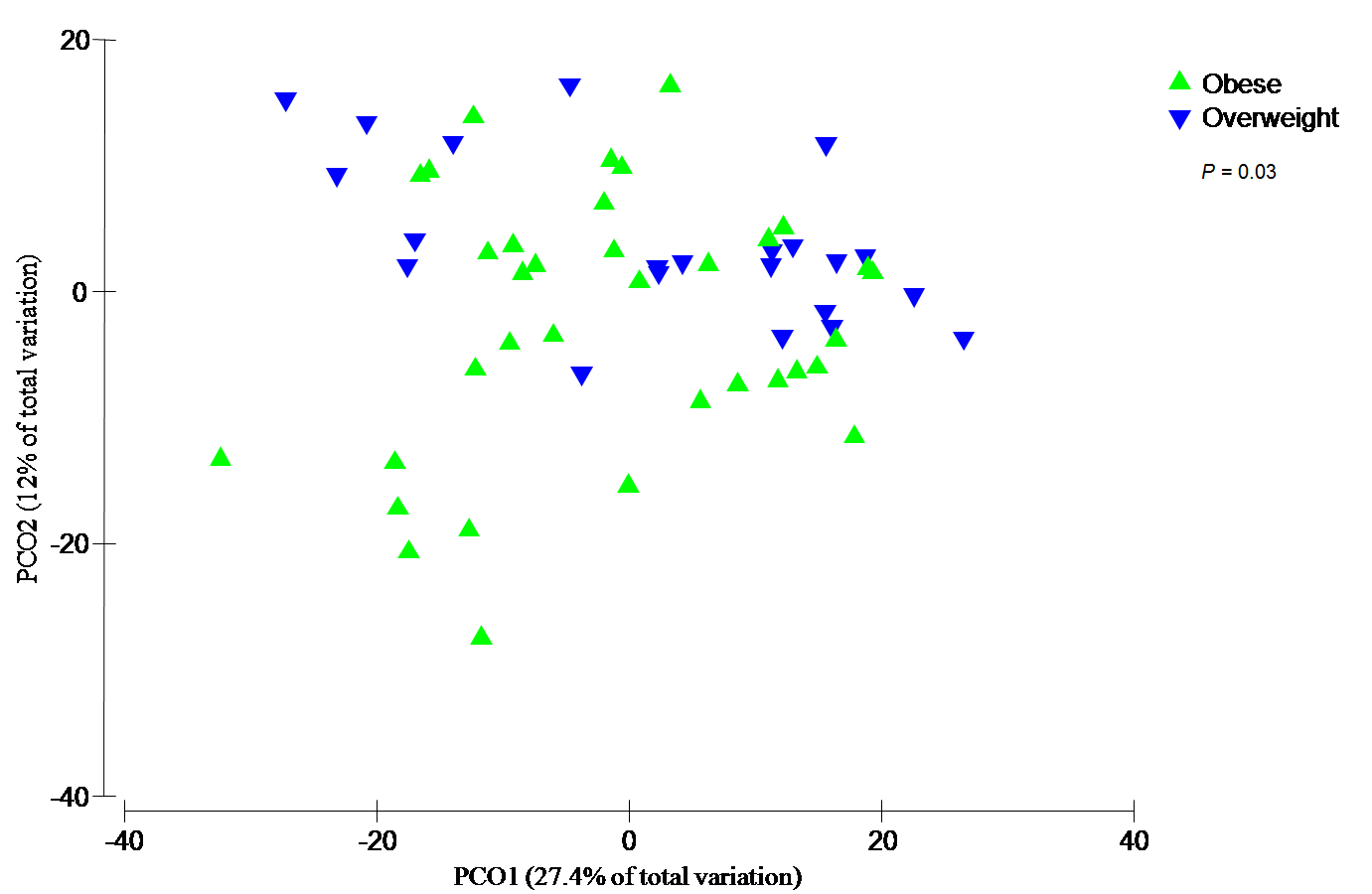

Supplement: FIGURE S4 — Principal coordinates analysis (PCoA) of the Bray-Curtis distance between the overweight and obese subjects. [file Image_4.TIF]

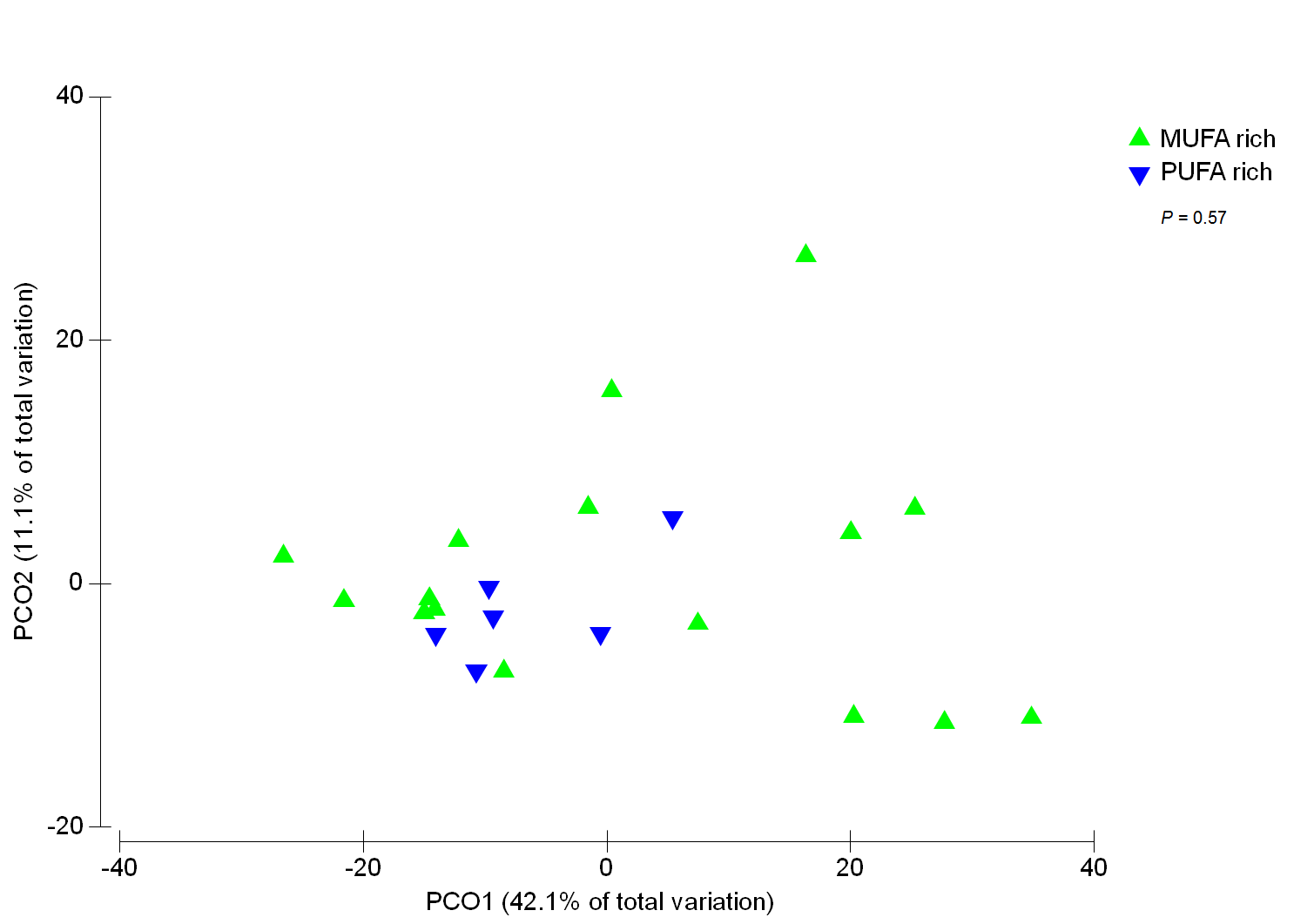

Supplement: FIGURE S5 — Principal coordinates analysis (PCoA) of the Bray-Curtis distance between the MUFA and PUFA oil treatments within overweight group. [file Image_5.TIF]

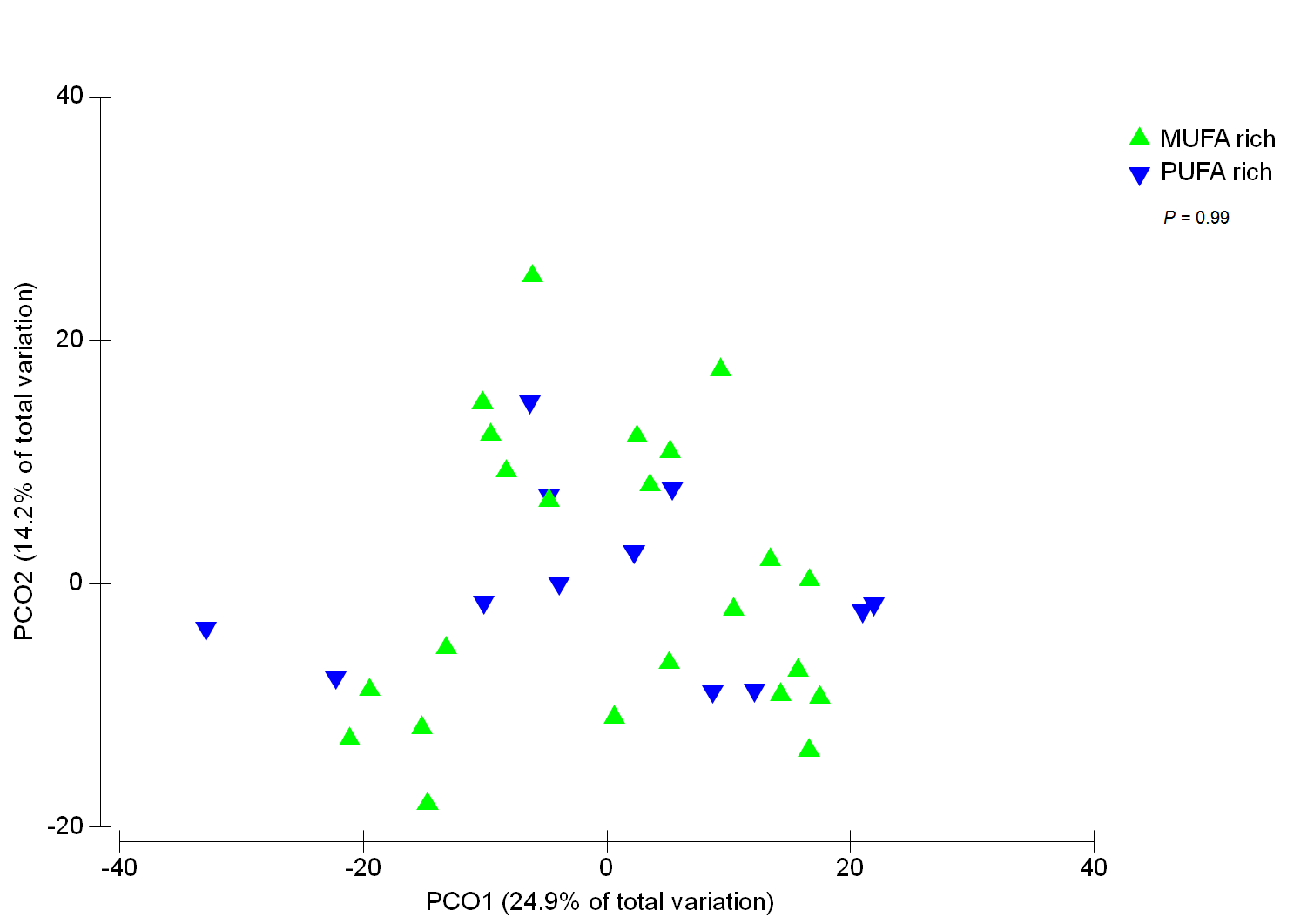

Supplement: FIGURE S6 — Principal coordinates analysis (PCoA) of the Bray-Curtis distance between the MUFA and PUFA oil treatments within obese group. [file Image_6.TIF]

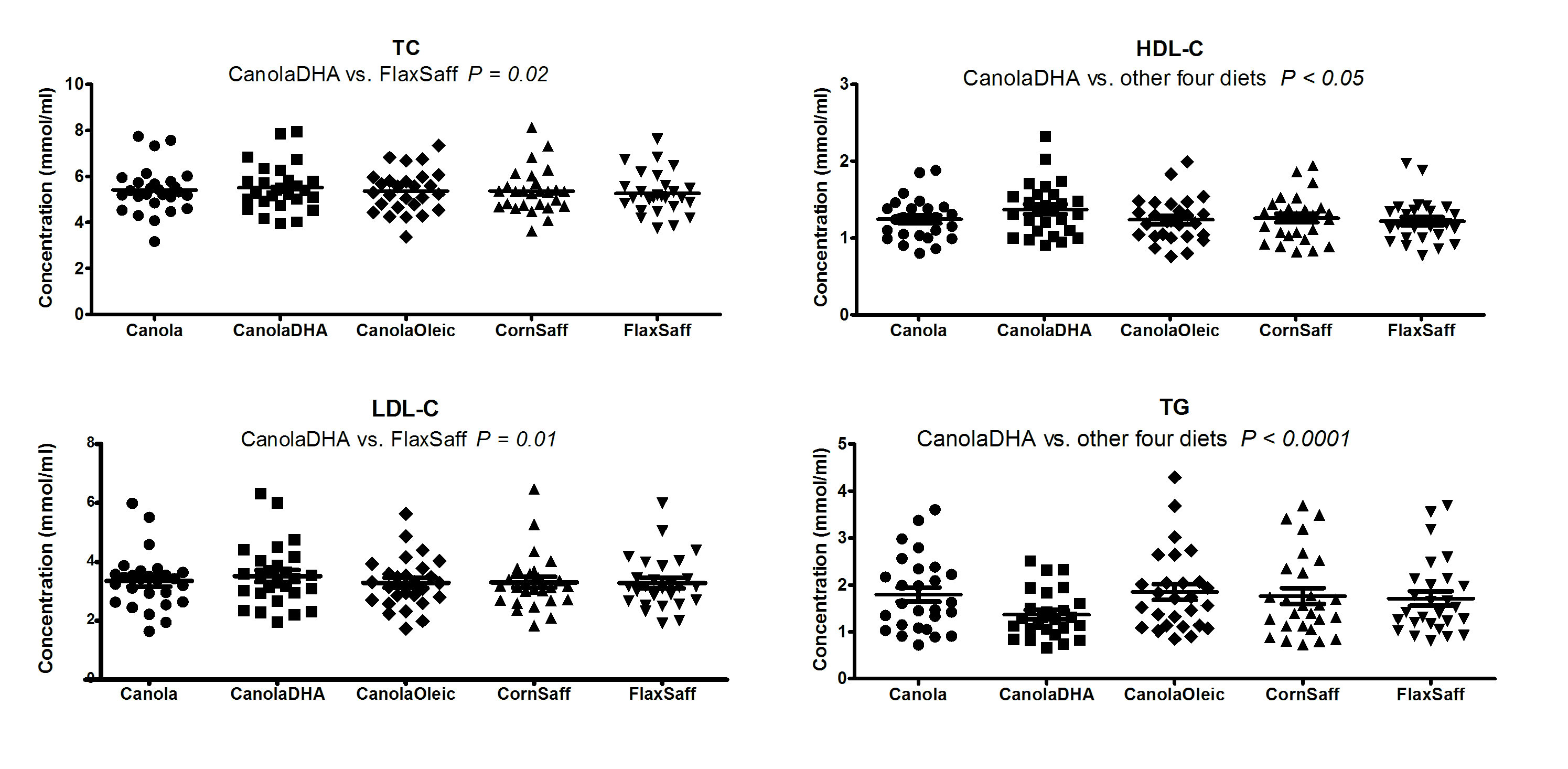

Supplement: FIGURE S7 — Serum lipid profiles at the endpoint of five experimental diets. Significant differences between treatments are highlighted within each parameter. [file Image_7.TIF]
