# Supplementary material for: Interactions between Obesity Status and Dietary Intake of Monounsaturated and Polyunsaturated Oils on Human Gut Microbiome Profiles in the Canola Oil Multicenter Intervention Trial (COMIT)
Source: Front Microbiol. 2016 Oct 10;7:1612. doi: 10.3389/fmicb.2016.01612 (PMC5056191; doi:10.3389/fmicb.2016.01612)
Supplement: TABLE S1 — List of fecal samples collected from the COMIT study. [file Table_1.DOCX]

| Table S1. List of fecal samples collected from the COMIT study | | | | | |  |
| --- | --- | --- | --- | --- | --- | --- |
| Subject ID | Treatments^1^ | | | | | BMI category^2^ |
|  | Canola | CanolaDHA | CanolaOleic | CornSaff | FlaxSaff |  |
| 100 | y | y |  |  |  | Obese |
| 101 |  | y | y | y | y | Overweight |
| 103 | y | y |  |  |  | Overweight |
| 104 |  | y | y |  |  | Overweight |
| 105 | y | y |  |  | y | Obese |
| 109 | y |  |  |  |  | Normal |
| 110 | y |  | y |  |  | Overweight |
| 112 | y | y | y |  |  | Overweight |
| 113 |  |  | y | y |  | Obese |
| 115 | y |  | y | y |  | Obese |
| 116 | y |  | y |  |  | Obese |
| 117 |  | y |  |  |  | Overweight |
| 118 |  | y | y |  | y | Obese |
| 123 | y |  |  | y |  | Overweight |
| 124 | y |  | y | y |  | Obese |
| 125 | y |  |  |  | y | Overweight |
| 126 | y |  |  | y | y | Obese |
| 129 | y |  |  | y |  | Obese |
| 130 |  | y | y | y | y | Normal |
| 131 |  |  | y | y |  | Obese |
| 132 | y | y |  | y | y | Overweight |
| 134 | y | y |  | y | y | Obese |
| 135 | y | y | y |  |  | Obese |
| 136 |  | y | y | y |  | Obese |
| 137 | y | y |  | y | y | Normal |
| Total | 17 | 14 | 13 | 13 | 9 |  |
| ^1^Dietary oil treatments are Canola: conventional canola oil; CanolaDHA: high oleic canola oil with DHA (85:15); CanolaOleic: high oleic canola oil; CornSaff: corn oil and safflower oil blend (25:75); FlaxSaff: flax oil and safflower oil blend (60:40). | | | | | | |
| ^2^Twenty-five participants in three different BMI categories: Normal (BMI≤25, n=3), overweight (25<BMI≤30, n=9), and obese (BMI>30, n=13). | | | | | | |
