# Supplementary material for: Interactions between Obesity Status and Dietary Intake of Monounsaturated and Polyunsaturated Oils on Human Gut Microbiome Profiles in the Canola Oil Multicenter Intervention Trial (COMIT)
Source: Front Microbiol. 2016 Oct 10;7:1612. doi: 10.3389/fmicb.2016.01612 (PMC5056191; doi:10.3389/fmicb.2016.01612)
Supplement: TABLE S2 — Example of a 7-day rotation isocaloric menu during the intervention. [file Table_2.DOCX]

| Table S2. Example of 7-day rotation isocaloric menu during the intervention | | | | | | | | |
| --- | --- | --- | --- | --- | --- | --- | --- | --- |
|  | Calories (kcal) | Protein (g) | Carbohydrate (g) | Fat (g) | MUFA (g) | PUFA (g) | SFA (g) | Fiber (g) |
| **Day 1** |  |  |  |  |  |  |  |  |
| Breakfast | 742.56 | 23.35 | 78.00 | 38.41 | 20.84 | 8.68 | 7.60 | 5.22 |
| Lunch | 592.24 | 30.32 | 81.79 | 17.50 | 5.76 | 4.47 | 5.68 | 12.37 |
| Dinner | 1020.58 | 52.18 | 134.33 | 29.95 | 10.91 | 6.73 | 8.95 | 13.08 |
| Evening snack | 650.87 | 8.01 | 79.05 | 34.73 | 20.12 | 9.73 | 3.96 | 4.66 |
| **Day 1 total** | **3006.25** | **113.86** | **373.17** | **120.59** | **57.63** | **29.61** | **26.19** | **35.33** |
| **Day 2** |  |  |  |  |  |  |  |  |
| Breakfast | 778.84 | 14.97 | 105.86 | 35.88 | 19.67 | 8.59 | 6.25 | 8.63 |
| Lunch | 699.22 | 38.69 | 109.38 | 14.33 | 5.19 | 5.18 | 2.43 | 19.54 |
| Dinner | 892.82 | 56.63 | 87.67 | 35.26 | 14.92 | 6.90 | 10.42 | 8.53 |
| Evening snack | 606.12 | 6.86 | 71.40 | 33.62 | 20.16 | 8.49 | 4.14 | 4.17 |
| **Day 2 total** | **2977.00** | **117.15** | **374.31** | **119.09** | **59.94** | **29.16** | **23.24** | **40.87** |
| **Day 3** |  |  |  |  |  |  |  |  |
| Breakfast | 841.31 | 33.75 | 84.42 | 42.75 | 22.24 | 9.33 | 9.18 | 9.30 |
| Lunch | 731.92 | 35.30 | 127.16 | 13.25 | 3.03 | 5.40 | 2.49 | 15.70 |
| Dinner | 737.61 | 38.42 | 90.46 | 26.14 | 9.59 | 6.45 | 7.61 | 8.18 |
| Evening snack | 683.52 | 8.35 | 81.54 | 37.09 | 22.11 | 8.95 | 5.01 | 3.95 |
| **Day 3 total** | **2994.36** | **115.82** | **383.58** | **119.23** | **56.97** | **30.13** | **24.29** | **37.13** |
| **Day 4** |  |  |  |  |  |  |  |  |
| Breakfast | 794.76 | 18.28 | 101.41 | 39.01 | 21.24 | 9.88 | 7.20 | 11.27 |
| Lunch | 819.66 | 46.12 | 113.73 | 19.79 | 9.14 | 4.66 | 4.55 | 16.02 |
| Dinner | 792.96 | 50.15 | 85.19 | 26.43 | 10.56 | 4.98 | 7.59 | 10.66 |
| Evening snack | 605.33 | 10.20 | 68.89 | 33.19 | 18.95 | 8.39 | 4.76 | 4.37 |
| **Day 4 total** | **3012.71** | **124.75** | **369.22** | **118.42** | **59.89** | **27.91** | **24.10** | **42.32** |
| **Day 5** |  |  |  |  |  |  |  |  |
| Breakfast | 765.00 | 12.36 | 99.78 | 38.51 | 21.79 | 10.62 | 4.86 | 11.16 |
| Lunch | 731.23 | 42.86 | 129.98 | 5.89 | 2.47 | 1.47 | 1.29 | 13.60 |
| Dinner | 672.42 | 41.86 | 73.43 | 25.07 | 10.42 | 6.14 | 6.04 | 9.75 |
| Evening snack | 827.27 | 15.54 | 84.92 | 49.85 | 27.38 | 13.98 | 7.11 | 6.46 |
| **Day 5 total** | **2995.92** | **112.62** | **388.11** | **119.32** | **62.06** | **32.21** | **19.30** | **40.97** |
| **Day 6** |  |  |  |  |  |  |  |  |
| Breakfast | 904.16 | 35.81 | 100.60 | 42.32 | 22.12 | 9.37 | 8.71 | 12.68 |
| Lunch | 682.13 | 33.21 | 107.53 | 14.86 | 1.75 | 0.72 | 5.15 | 5.65 |
| Dinner | 752.43 | 33.15 | 110.96 | 19.28 | 6.45 | 5.55 | 4.26 | 19.45 |
| Evening snack | 654.56 | 10.70 | 63.35 | 42.54 | 23.54 | 12.06 | 5.61 | 4.66 |
| **Day 6 total** | **2993.28** | **112.87** | **382.44** | **119.00** | **53.86** | **27.70** | **23.73** | **42.44** |
| **Day 7** |  |  |  |  |  |  |  |  |
| Breakfast | 756.73 | 14.10 | 104.96 | 34.20 | 19.23 | 8.52 | 5.25 | 8.63 |
| Lunch | 756.49 | 30.57 | 113.47 | 22.53 | 11.71 | 7.02 | 3.35 | 14.77 |
| Dinner | 806.58 | 65.11 | 79.65 | 24.19 | 9.80 | 6.79 | 4.76 | 9.33 |
| Evening snack | 683.52 | 8.35 | 81.54 | 37.09 | 22.11 | 8.95 | 5.01 | 3.95 |
| **Day 7 total** | **3003.32** | **118.13** | **379.62** | **118.01** | **62.85** | **31.28** | **18.37** | **36.68** |
| **7-day average** | **2997.55** | **116.46** | **378.64** | **119.09** | **59.03** | **29.71** | **22.75** | **39.39** |
| A fixed 7-day rotation isocaloric menu of three meals and snacks, including 50% carbohydrate 15% protein and 35% fat of total energy of 3000 Kcal per day were designed for the interventions. The daily 60-g dietary oil treatment was freshly made according to the required macronutrient of the menu, and equally distributed to two identical sizes of beverage shakes for morning and evening consumptions. This example presents the 7-day menu of a female subject in the COMIT study (age: 50 y; height: 5 ft. 5 in.; weight: 175.00 lb; BMI: 29.12). | | | | | | | | |
