# Supplementary material for: Interactions between Obesity Status and Dietary Intake of Monounsaturated and Polyunsaturated Oils on Human Gut Microbiome Profiles in the Canola Oil Multicenter Intervention Trial (COMIT)
Source: Front Microbiol. 2016 Oct 10;7:1612. doi: 10.3389/fmicb.2016.01612 (PMC5056191; doi:10.3389/fmicb.2016.01612)
Supplement: TABLE S3 — Summary of sequenced OTUs at each taxonomic level. [file Table_3.DOCX]

| Table S3. Summary of sequenced OTUs at each taxonomic level | | | | | |
| --- | --- | --- | --- | --- | --- |
|  | Phylum | Class | Order | Family | Genus |
| Classified | 2189 | 2180 | 2166 | 2095 | 1592 |
| Unclassified | 4 | 13 | 27 | 98 | 601 |
| % Sequenced | 99.82 | 99.41 | 98.77 | 95.53 | 72.59 |
