# Supplementary material for: Interactions between Obesity Status and Dietary Intake of Monounsaturated and Polyunsaturated Oils on Human Gut Microbiome Profiles in the Canola Oil Multicenter Intervention Trial (COMIT)
Source: Front Microbiol. 2016 Oct 10;7:1612. doi: 10.3389/fmicb.2016.01612 (PMC5056191; doi:10.3389/fmicb.2016.01612)
Supplement: TABLE S4 — Phylogenetic composition of putative bacterial genera (above 0.1% of community) determined using 16S rRNA gene pyrosequencing. [file Table_4.DOCX]

| Table S4. Phylogenetic composition of putative bacterial genera (above 0.1% of community) determined using 16S rRNA pyrosequencing | | | | | | | | | | | |  |  |  |  |
| --- | --- | --- | --- | --- | --- | --- | --- | --- | --- | --- | --- | --- | --- | --- | --- |
| Taxa (phylum, family, genus)^1^ | | | Pecentages of Sequences in Treatments^2^ | | | | | Mean | SEM | *P*-value^3^ | Contrast (*P*-value)^4^ | | | | |
|  |  |  | Canola | CanolaDHA | CanolaOleic | CornSaff | FlaxSaff |  |  |  | MUFA/ | CanolaDHA/ | CornSaff/ | CornSaff/ | FlaxSaff/ |
|  |  |  |  |  |  |  |  |  |  |  | PUFA | CanolaOleic | FlaxSaff | CanolaOleic | CanolaOleic |
| Actinobacteria | | |  |  |  |  |  |  |  |  |  |  |  |  |  |
|  | Coriobacteriaceae | |  |  |  |  |  |  |  |  |  |  |  |  |  |
|  |  | *Adlercreutzia* | 1.33 | 1.58 | 0.92 | 1.01 | 1.01 | 1.20 | 0.11 | 0.59 | 0.42 | 0.62 | 1.00 | 1.00 | 1.00 |
|  |  | *Collinsella* | 0.77 | 1.08 | 0.90 | 0.67 | 0.65 | 0.82 | 0.09 | 0.80 | 0.34 | 0.99 | 1.00 | 0.97 | 0.97 |
|  |  | *Coriobacterium* | 0.09 | 0.15 | 0.22 | 0.30 | 0.18 | 0.18 | 0.05 | 0.94 | 0.49 | 1.00 | 1.00 | 1.00 | 0.82 |
|  |  | *Slackia* | 0.23 | 0.08 | 0.20 | 0.37 | 0.08 | 0.20 | 0.06 | 0.68 | 0.57 | 0.98 | 0.81 | 0.94 | 0.98 |
| Aquificae | | |  |  |  |  |  |  |  |  |  |  |  |  |  |
|  | Aquificaceae | |  |  |  |  |  |  |  |  |  |  |  |  |  |
|  |  | *Hydrogenobaculum* | 0.58 | 0.63 | 0.43 | 0.76 | 0.26 | 0.55 | 0.12 | 0.79 | 0.97 | 0.92 | 0.90 | 0.96 | 1.00 |
| Bacteroidetes | | |  |  |  |  |  |  |  |  |  |  |  |  |  |
|  | Bacteroidaceae | |  |  |  |  |  |  |  |  |  |  |  |  |  |
|  |  | *Bacteroides* | 6.02 | 5.24 | 7.18 | 5.37 | 5.09 | 5.83 | 0.45 | 0.94 | 0.59 | 0.94 | 0.48 | 1.00 | 0.50 |
|  | Cyclobacteriaceae | |  |  |  |  |  |  |  |  |  |  |  |  |  |
|  |  | *Aquiflexum* | 0.27 | 0.13 | 0.15 | 0.04 | 0.20 | 0.16 | 0.06 | 0.63 | 0.39 | 1.00 | 0.75 | 0.90 | 0.99 |
|  | Marinilabiaceae | |  |  |  |  |  |  |  |  |  |  |  |  |  |
|  |  | *Anaerophaga* | 0.16 | 0.26 | 0.07 | 0.41 | 0.20 | 0.22 | 0.06 | 0.75 | 0.42 | 0.95 | 0.93 | 0.80 | 0.99 |
|  | Porphyromonadaceae | | |  |  |  |  |  |  |  |  |  |  |  |  |
|  |  | *Barnesiella* | 0.42 | 0.37 | 0.38 | 0.25 | 0.34 | 0.36 | 0.07 | 0.91 | 0.59 | 0.99 | 0.97 | 1.00 | 1.00 |
|  |  | *Parabacteroides* | 0.95 | 1.23 | 1.54 | 0.65 | 0.61 | 1.02 | 0.12 | 0.35 | 0.09 | 0.99 | 1.00 | 0.36 | 0.69 |
|  | Rikenellaceae | |  |  |  |  |  |  |  |  |  |  |  |  |  |
|  |  | *Alistipes* | 2.94 | 3.42 | 2.36 | 2.91 | 2.79 | 2.90 | 0.21 | 0.53 | 0.43 | 0.54 | 0.99 | 1.00 | 0.98 |
| Firmicutes | | |  |  |  |  |  |  |  |  |  |  |  |  |  |
|  | Carnobacteriaceae | |  |  |  |  |  |  |  |  |  |  |  |  |  |
|  |  | *Granulicatella* | 0.13 | 0.16 | 0.05 | 0.06 | 0.20 | 0.12 | 0.05 | 0.87 | 0.98 | 0.94 | 0.93 | 1.00 | 0.91 |
|  |  | *Isobaculum* | 0.28 | 0.45 | 0.51 | 0.91 | 0.62 | 0.53 | 0.10 | 0.30 | 0.08 | 0.99 | 0.83 | 0.83 | 1.00 |
|  |  | *Marinilactibacillus* | 0.27 | 0.46 | 0.91 | 0.65 | 0.58 | 0.55 | 0.11 | 0.31 | 0.57 | 0.63 | 0.99 | 0.99 | 0.91 |
|  | Clostridiaceae | |  |  |  |  |  |  |  |  |  |  |  |  |  |
|  |  | *Clostridium* | 0.46 | 0.66 | 0.56 | 0.40 | 0.94 | 0.58 | 0.08 | 0.57 | 0.79 | 1.00 | 0.59 | 0.97 | 0.90 |
|  |  | *Geosporobacter* | 0.45 | 0.04 | 0.07 | 0.14 | 0.48 | 0.23 | 0.08 | 0.32 | 0.86 | 0.98 | 0.76 | 1.00 | 0.80 |
|  | Erysipelotrichaceae | |  |  |  |  |  |  |  |  |  |  |  |  |  |
|  |  | *Allobaculum* | 0.52 | 0.59 | 0.60 | 0.50 | 0.52 | 0.54 | 0.10 | 0.98 | 0.61 | 1.00 | 1.00 | 1.00 | 0.99 |
|  |  | *Coprobacillus* | 1.71 | 1.15 | 2.16 | 1.93 | 1.72 | 1.73 | 0.13 | 0.38 | 0.60 | 0.28 | 1.00 | 0.99 | 0.95 |
|  |  | *Turicibacter* | 0.34 | 0.48 | 0.40 | 0.21 | 0.16 | 0.33 | 0.05 | 0.66 | 0.17 | 1.00 | 1.00 | 0.90 | 0.86 |
|  | Gracilibacteraceae | |  |  |  |  |  |  |  |  |  |  |  |  |  |
|  |  | *Gracilibacter* | 0.32 | 0.12 | 0.12 | 0.05 | 0.21 | 0.17 | 0.06 | 0.59 | 0.51 | 1.00 | 0.88 | 0.98 | 0.99 |
|  | Heliobacteriaceae | |  |  |  |  |  |  |  |  |  |  |  |  |  |
|  |  | *Heliorestis* | 0.16 | 0.12 | 0.42 | 0.32 | 1.05 | 0.35 | 0.14 | 0.20 | 0.17 | 0.52 | 0.55 | 0.97 | 0.88 |
|  | Incertae_Sedis_XI | |  |  |  |  |  |  |  |  |  |  |  |  |  |
|  |  | *Anaerococcus* | 1.76 | 1.65 | 1.27 | 1.56 | 2.22 | 1.66 | 0.22 | 0.99 | 0.75 | 1.00 | 0.99 | 1.00 | 0.99 |
|  |  | *Soehngenia* | 2.23 | 2.16 | 1.94 | 2.42 | 1.20 | 2.05 | 0.25 | 0.74 | 0.90 | 0.96 | 0.84 | 0.98 | 0.98 |
|  |  | *Tepidimicrobium* | 0.17 | 0.51 | 0.04 | 0.13 | 0.20 | 0.21 | 0.07 | 0.29 | 0.62 | 0.51 | 1.00 | 0.95 | 0.87 |
|  |  | *Tissierella* | 1.73 | 1.38 | 0.75 | 1.54 | 1.41 | 1.38 | 0.26 | 0.57 | 0.77 | 1.00 | 0.97 | 0.85 | 1.00 |
|  | Incertae_Sedis_XII | |  |  |  |  |  |  |  |  |  |  |  |  |  |
|  |  | unclassified | 0.19 | 0.75 | 0.31 | 0.17 | 0.63 | 0.39 | 0.09 | 0.12 | 0.67 | 0.67 | 0.55 | 0.92 | 0.94 |
|  | Incertae_Sedis_XIII | | |  |  |  |  |  |  |  |  |  |  |  |  |
|  |  | *Anaerovorax* | 0.83 | 0.64 | 0.96 | 0.85 | 0.59 | 0.79 | 0.08 | 0.85 | 0.77 | 0.89 | 0.96 | 1.00 | 0.88 |
|  |  | *Mogibacterium* | 0.09 | 0.30 | 0.25 | 0.18 | 0.33 | 0.22 | 0.06 | 0.55 | 0.89 | 0.92 | 0.79 | 1.00 | 0.92 |
|  | Incertae_Sedis_XIV | | |  |  |  |  |  |  |  |  |  |  |  |  |
|  |  | *Blautia* | 7.73 | 6.50 | 9.05 | 7.34 | 7.10 | 7.57 | 0.49 | 0.11 | 0.25 | 0.09 | 1.00 | 0.98 | 0.99 |
|  | Lachnospiraceae | |  |  |  |  |  |  |  |  |  |  |  |  |  |
|  |  | *Coprococcus* | 4.75 | 4.96 | 3.74 | 4.76 | 5.26 | 4.67 | 0.33 | 0.50 | 0.29 | 0.21 | 0.92 | 0.10 | 0.18 |
|  |  | *Dorea* | 2.29 | 2.36 | 2.66 | 2.22 | 2.25 | 2.36 | 0.24 | 0.85 | 0.72 | 0.98 | 0.96 | 0.97 | 1.00 |
|  |  | *Hespellia* | 2.28 | 2.31 | 1.54 | 2.09 | 1.80 | 2.04 | 0.21 | 0.38 | 0.20 | 0.82 | 0.97 | 1.00 | 1.00 |
|  |  | *Parasporobacterium* | 1.27 | 1.29 | 1.62 | 1.84 | 1.92 | 1.54 | 0.16 | 0.76 | 0.38 | 0.94 | 1.00 | 0.98 | 1.00 |
|  |  | *Pseudobutyrivibrio* | 2.11 | 1.65 | 1.89 | 1.87 | 2.05 | 1.91 | 0.22 | 0.57 | 0.28 | 0.91 | 0.97 | 0.73 | 0.99 |
|  |  | *Roseburia* | 4.46 | 4.11 | 5.08 | 4.33 | 4.81 | 4.53 | 0.25 | 0.86 | 0.85 | 0.27 | 0.96 | 0.46 | 0.55 |
|  |  | *Sporobacterium* | 0.27 | 0.08 | 0.11 | 0.35 | 0.60 | 0.26 | 0.08 | 0.48 | 0.14 | 0.98 | 0.96 | 0.97 | 0.83 |
|  |  | *Syntrophococcus* | 0.10 | 0.13 | 0.23 | 0.12 | 0.12 | 0.14 | 0.04 | 0.94 | 0.85 | 0.98 | 1.00 | 0.98 | 0.99 |
|  |  | unclassified1 | 0.33 | 0.14 | 0.33 | 0.47 | 0.39 | 0.32 | 0.07 | 0.68 | 0.27 | 0.85 | 1.00 | 0.98 | 1.00 |
|  |  | unclassified2 | 18.96 | 19.25 | 17.18 | 19.35 | 20.17 | 18.91 | 0.83 | 0.55 | 0.43 | 0.77 | 0.24 | 0.13 | 0.84 |
|  | Lactobacillaceae | |  |  |  |  |  |  |  |  |  |  |  |  |  |
|  |  | *Lactobacillus* | 0.18 | 0.11 | 0.14 | 0.12 | 0.12 | 0.14 | 0.05 | 0.95 | 0.97 | 1.00 | 1.00 | 0.99 | 1.00 |
|  | Peptococcaceae | |  |  |  |  |  |  |  |  |  |  |  |  |  |
|  |  | unclassified | 0.13 | 0.14 | 0.11 | 0.21 | 0.10 | 0.14 | 0.04 | 0.98 | 0.88 | 1.00 | 0.98 | 0.99 | 1.00 |
|  | Peptostreptococcaceae | | |  |  |  |  |  |  |  |  |  |  |  |  |
|  |  | *Sporacetigenium* | 0.70 | 0.79 | 0.74 | 0.73 | 0.85 | 0.75 | 0.08 | 0.99 | 0.85 | 1.00 | 1.00 | 1.00 | 1.00 |
|  |  | unclassified | 0.74 | 0.96 | 0.61 | 0.81 | 1.05 | 0.82 | 0.11 | 0.82 | 0.58 | 0.82 | 1.00 | 0.94 | 0.81 |
|  | Ruminococcaceae | |  |  |  |  |  |  |  |  |  |  |  |  |  |
|  |  | *Butyricicoccus* | 0.94 | 1.25 | 0.92 | 1.15 | 1.33 | 1.10 | 0.12 | 0.81 | 0.35 | 0.97 | 1.00 | 0.96 | 0.87 |
|  |  | *Faecalibacterium* | 2.18 | 2.18 | 4.15 | 2.64 | 3.43 | 2.83 | 0.26 | 0.06 | 0.81 | 0.07 | 1.00 | 0.25 | 0.35 |
|  |  | *Oscillibacter* | 2.63 | 3.22 | 3.12 | 2.97 | 2.92 | 2.96 | 0.26 | 0.89 | 0.69 | 1.00 | 1.00 | 0.98 | 1.00 |
|  |  | *Papillibacter* | 2.03 | 1.64 | 1.77 | 2.20 | 2.03 | 1.93 | 0.17 | 0.92 | 0.62 | 1.00 | 1.00 | 0.97 | 0.99 |
|  |  | *Ruminococcus* | 0.35 | 0.22 | 0.35 | 0.49 | 0.60 | 0.38 | 0.06 | 0.66 | 0.18 | 0.96 | 1.00 | 0.98 | 0.92 |
|  |  | *Sporobacter* | 2.93 | 3.13 | 2.22 | 3.01 | 2.56 | 2.80 | 0.26 | 0.91 | 0.85 | 0.93 | 0.99 | 0.98 | 1.00 |
|  |  | *Subdoligranulum* | 2.28 | 1.94 | 1.15 | 2.07 | 1.71 | 1.87 | 0.16 | 0.35 | 0.76 | 0.57 | 0.99 | 0.44 | 0.80 |
|  |  | unclassified | 4.73 | 5.19 | 3.48 | 3.95 | 5.15 | 4.48 | 0.38 | 0.28 | 0.20 | 0.38 | 0.13 | 0.40 | 0.46 |
|  | Streptococcaceae | |  |  |  |  |  |  |  |  |  |  |  |  |  |
|  |  | *Streptococcus* | 1.56 | 1.52 | 1.63 | 1.02 | 1.04 | 1.39 | 0.15 | 0.78 | 0.19 | 1.00 | 1.00 | 0.88 | 0.95 |
|  | Veillonellaceae | |  |  |  |  |  |  |  |  |  |  |  |  |  |
|  |  | *Dialister* | 0.46 | 0.62 | 0.76 | 0.20 | 0.39 | 0.49 | 0.10 | 0.64 | 0.22 | 1.00 | 0.99 | 0.71 | 0.96 |
|  | unclassified | |  |  |  |  |  |  |  |  |  |  |  |  |  |
|  |  | unclassified1 | 0.17 | 0.13 | 0.20 | 0.25 | 0.31 | 0.20 | 0.05 | 0.90 | 0.38 | 0.99 | 1.00 | 1.00 | 0.99 |
|  |  | unclassified2 | 0.34 | 0.24 | 0.24 | 0.30 | 0.15 | 0.27 | 0.08 | 0.92 | 0.65 | 1.00 | 0.92 | 1.00 | 0.94 |
|  |  | unclassified3 | 1.27 | 1.69 | 0.59 | 0.98 | 1.07 | 1.14 | 0.15 | 0.19 | 0.28 | 0.20 | 1.00 | 0.96 | 0.92 |
| Proteobacteria | | |  |  |  |  |  |  |  |  |  |  |  |  |  |
|  | Alcaligenaceae | |  |  |  |  |  |  |  |  |  |  |  |  |  |
|  |  | *Sutterella* | 0.27 | 0.21 | 0.23 | 0.37 | 0.16 | 0.25 | 0.06 | 0.89 | 0.73 | 1.00 | 0.90 | 0.97 | 1.00 |
|  | Enterobacteriaceae | |  |  |  |  |  |  |  |  |  |  |  |  |  |
|  |  | *Escherichia/Shigella* | 0.17 | 0.34 | 0.29 | 0.16 | 0.45 | 0.26 | 0.05 | 0.67 | 0.92 | 1.00 | 0.75 | 0.95 | 0.98 |
|  | Rhodospirillaceae | |  |  |  |  |  |  |  |  |  |  |  |  |  |
|  |  | *Inquilinus* | 0.14 | 0.05 | 0.27 | 0.27 | 0.25 | 0.19 | 0.06 | 0.80 | 0.45 | 0.87 | 1.00 | 1.00 | 1.00 |
| Verrucomicrobia | | |  |  |  |  |  |  |  |  |  |  |  |  |  |
|  | Verrucomicrobiaceae | | |  |  |  |  |  |  |  |  |  |  |  |  |
|  |  | *Akkermansia* | 0.19 | 0.12 | 0.16 | 0.09 | 0.18 | 0.15 | 0.04 | 0.96 | 0.76 | 1.00 | 0.98 | 0.99 | 1.00 |
| ^1^Taxa are reported at phylum, family and genus levels. If the sequence is unable to classify, it is named unclassified. Labeled numbers in different unclassified genera indicate different genera within the same family. | | | | | | | | | | | | | | | |
| ^2^Dietary oil treatments are Canola: conventional canola oil; CanolaDHA: high oleic canola oil with DHA (85:15); CanolaOleic: high oleic canola oil; CornSaff: corn oil and safflower oil blend (25:75); FlaxSaff: flax oil and safflower oil blend (60:40). | | | | | | | | | | | | | | | |
| ^3^*P*-values for overall differences between five dietary oil treatments were analyzed by using linear mixed-model ANOVA with Tukey adjustment (*P*<0.05). | | | | | | | | | | | | |  |  |  |
| ^4^*P*-values for difference between all selected contrasts were analyzed by using linear mixed-model ANOVA with Tukey adjustment (*P*<0.05). | | | | | | | | | | | |  |  |  |  |
