# Supplementary material for: Interactions between Obesity Status and Dietary Intake of Monounsaturated and Polyunsaturated Oils on Human Gut Microbiome Profiles in the Canola Oil Multicenter Intervention Trial (COMIT)
Source: Front Microbiol. 2016 Oct 10;7:1612. doi: 10.3389/fmicb.2016.01612 (PMC5056191; doi:10.3389/fmicb.2016.01612)
Supplement: TABLE S5 — List of bacterial genera in low abundance (below 0.1% of community). [file Table_5.DOCX]

| Table S5. List of bacterial genera in low abundance (below 0.1% of community) | | |  |  |
| --- | --- | --- | --- | --- |
| Taxa (phylum, family, genus)^1^ | |  |  |  |
| Acidobacteria | unclassified | unclassified |  |  |
| Actinobacteria | Actinomycetaceae | *Actinomyces* |  |  |
| Actinobacteria | Micrococcaceae | *Sinomonas* |  |  |
| Actinobacteria | Pseudonocardiaceae | *Prauserella* |  |  |
| Actinobacteria | Bifidobacteriaceae | *Bifidobacterium* |  |  |
| Actinobacteria | Bifidobacteriaceae | *Gardnerella* |  |  |
| Actinobacteria | Coriobacteriaceae | *Atopobium* |  |  |
| Actinobacteria | Coriobacteriaceae | *Olsenella* |  |  |
| Aquificae | Aquificaceae | *Aquifex* |  |  |
| Aquificae | Desulfurobacteriaceae | *Thermovibrio* |  |  |
| Bacteroidetes | Marinifilum | unclassified |  |  |
| Bacteroidetes | Porphyromonadaceae | *Dysgonomonas* |  |  |
| Bacteroidetes | Porphyromonadaceae | *Petrimonas* |  |  |
| Bacteroidetes | Prevotellaceae | *Paraprevotella* |  |  |
| Bacteroidetes | Prevotellaceae | unclassified |  |  |
| Bacteroidetes | unclassified | unclassified |  |  |
| Bacteroidetes | Flavobacteriaceae | *Coenonia* |  |  |
| Bacteroidetes | Flavobacteriaceae | *Flavobacterium* |  |  |
| Bacteroidetes | Flavobacteriaceae | *Zhouia* |  |  |
| Chrysiogenetes | Chrysiogenaceae | *Chrysiogenes* |  |  |
| Cyanobacteria | Streptophyta | unclassified |  |  |
| Deinococcus-Thermus | Thermaceae | *Oceanithermus* |  |  |
| Firmicutes | Paenibacillaceae | *Cohnella* |  |  |
| Firmicutes | Paenibacillaceae | *Oxalophagus* |  |  |
| Firmicutes | Sporolactobacillaceae | *Tuberibacillus* |  |  |
| Firmicutes | Staphylococcaceae | *Gemella* |  |  |
| Firmicutes | Staphylococcaceae | *Staphylococcus* |  |  |
| Firmicutes | Thermoactinomycetaceae | *Thermoflavimicrobium* |  |  |
| Firmicutes | Carnobacteriaceae | *Allofustis* |  |  |
| Firmicutes | Carnobacteriaceae | *Desemzia* |  |  |
| Firmicutes | Carnobacteriaceae | *Dolosigranulum* |  |  |
| Firmicutes | Carnobacteriaceae | unclassified |  |  |
| Firmicutes | Enterococcaceae | *Atopobacter* |  |  |
| Firmicutes | Enterococcaceae | *Enterococcus* |  |  |
| Firmicutes | Enterococcaceae | *Melissococcus* |  |  |
| Firmicutes | Lactobacillaceae | *Lactococcus* |  |  |
| Firmicutes | unclassified1 | unclassified |  |  |
| Firmicutes | Clostridiaceae | *Natronincola* |  |  |
| Firmicutes | Clostridiaceae | *Thermohalobacter* |  |  |
| Firmicutes | Eubacteriaceae | *Alkalibacter* |  |  |
| Firmicutes | Eubacteriaceae | *Eubacterium* |  |  |
| Firmicutes | Incertae_Sedis_XI | *Finegoldia* |  |  |
| Firmicutes | Incertae_Sedis_XI | *Helcococcus* |  |  |
| Firmicutes | Incertae_Sedis_XI | *Parvimonas* |  |  |
| Firmicutes | Incertae_Sedis_XI | *Peptoniphilus* |  |  |
| Firmicutes | Incertae_Sedis_XI | unclassified |  |  |
| Firmicutes | Incertae_Sedis_XII | *Guggenheimella* |  |  |
| Firmicutes | Incertae_Sedis_XIV | *Howardella* |  |  |
| Firmicutes | Lachnospiraceae | *Catonella* |  |  |
| Firmicutes | Lachnospiraceae | *Moryella* |  |  |
| Firmicutes | Peptococcaceae | *Desulfurispora* |  |  |
| Firmicutes | Peptococcaceae | *Peptococcus* |  |  |
| Firmicutes | Peptococcaceae | *Thermincola* |  |  |
| Firmicutes | Ruminococcaceae | *Acetanaerobacterium* |  |  |
| Firmicutes | Ruminococcaceae | *Acetivibrio* |  |  |
| Firmicutes | Ruminococcaceae | *Ethanoligenens* |  |  |
| Firmicutes | Ruminococcaceae | unclassified |  |  |
| Firmicutes | Veillonellaceae | *Acidaminococcus* |  |  |
| Firmicutes | Veillonellaceae | *Anaeroarcus* |  |  |
| Firmicutes | Veillonellaceae | *Anaeroglobus* |  |  |
| Firmicutes | Veillonellaceae | *Anaeromusa* |  |  |
| Firmicutes | Veillonellaceae | *Anaerosinus* |  |  |
| Firmicutes | Veillonellaceae | *Megasphaera* |  |  |
| Firmicutes | Veillonellaceae | *Mitsuokella* |  |  |
| Firmicutes | Veillonellaceae | *Succinispira* |  |  |
| Firmicutes | Veillonellaceae | *Veillonella* |  |  |
| Firmicutes | Veillonellaceae | unclassified |  |  |
| Firmicutes | Thermoanaerobacteraceae | *Carboxydibrachium* |  |  |
| Firmicutes | Thermoanaerobacteraceae | *Mahella* |  |  |
| Firmicutes | Erysipelotrichaceae | *Bulleidia* |  |  |
| Firmicutes | Erysipelotrichaceae | *Holdemania* |  |  |
| Firmicutes | unclassified2 | unclassified |  |  |
| Nitrospira | Nitrospiraceae | unclassified |  |  |
| Proteobacteria | Caulobacteraceae | unclassified |  |  |
| Proteobacteria | Hyphomonadaceae | unclassified |  |  |
| Proteobacteria | Rhodobiaceae | *Parvibaculum* |  |  |
| Proteobacteria | Rhodobacteraceae | *Maritimibacter* |  |  |
| Proteobacteria | Rhodobacteraceae | *Nereida* |  |  |
| Proteobacteria | Rhodobacteraceae | unclassified |  |  |
| Proteobacteria | Acetobacteraceae | *Saccharibacter* |  |  |
| Proteobacteria | Acetobacteraceae | *Swaminathania* |  |  |
| Proteobacteria | Rhodospirillaceae | *Telmatospirillum* |  |  |
| Proteobacteria | unclassified1 | unclassified |  |  |
| Proteobacteria | Burkholderiaceae | *Paucimonas* |  |  |
| Proteobacteria | Burkholderiaceae | *Ralstonia* |  |  |
| Proteobacteria | Comamonadaceae | *Acidovorax* |  |  |
| Proteobacteria | Comamonadaceae | *Pelomonas* |  |  |
| Proteobacteria | Comamonadaceae | *Pseudacidovorax* |  |  |
| Proteobacteria | Procabacteriaceae | *Procabacter* |  |  |
| Proteobacteria | Desulfovibrionaceae | *Desulfovibrio* |  |  |
| Proteobacteria | Desulfovibrionaceae | *Lawsonia* |  |  |
| Proteobacteria | Phaselicystidaceae | *Phaselicystis* |  |  |
| Proteobacteria | unclassified2 | unclassified |  |  |
| Proteobacteria | Nautiliaceae | unclassified |  |  |
| Proteobacteria | Succinivibrionaceae | *Anaerobiospirillum* |  |  |
| Proteobacteria | Chromatiaceae | *Rhabdochromatium* |  |  |
| Proteobacteria | Granulosicoccaceae | *Granulosicoccus* |  |  |
| Proteobacteria | Enterobacteriaceae | *Kluyvera* |  |  |
| Proteobacteria | Enterobacteriaceae | *Pantoea* |  |  |
| Proteobacteria | Enterobacteriaceae | *Proteus* |  |  |
| Proteobacteria | Enterobacteriaceae | *Raoultella* |  |  |
| Proteobacteria | Enterobacteriaceae | *Serratia* |  |  |
| Proteobacteria | Pasteurellaceae | *Haemophilus* |  |  |
| Proteobacteria | Pseudomonadaceae | unclassified |  |  |
| Synergistetes | Synergistaceae | *Cloacibacillus* |  |  |
| TM7 | unclassified | unclassified |  |  |
| Tenericutes | unclassified | unclassified |  |  |
| Verrucomicrobia | Verrucomicrobiaceae | *Persicirhabdus* |  |  |
| ^1^Taxa are reported at phylum, family and genus levels. If the sequence is unable to classify, it is named unclassified. Labeled numbers in different unclassified level indicate different unclassified families or genera. | | | | |
